# Prenatal Mercury Exposure and Neurodevelopment up to the Age of 5 Years: A Systematic Review

**DOI:** 10.3390/ijerph19041976

**Published:** 2022-02-10

**Authors:** Kyle Dack, Matthew Fell, Caroline M. Taylor, Alexandra Havdahl, Sarah J. Lewis

**Affiliations:** 1 Medical Research Council Integrative Epidemiology Unit, University of Bristol, Bristol BS8 1TH, UK; 2 Cleft Collective, University of Bristol, Bristol BS8 1TH, UK; mattfell@doctors.org.uk; 3 Centre for Academic Child Health, Bristol Medical School, University of Bristol, Bristol BS8 1TH, UK; Caroline.m.taylor@bristol.ac.uk; 4 Department of Mental Disorders, Norwegian Institute of Public Health, 0213 Oslo, Norway; Alexandra.havdahl@fhi.no; 5 Nic Waals Institute, Lovisenberg Diaconal Hospital, 0853 Oslo, Norway; 6 Population Health Sciences, Bristol Medical School, University of Bristol, Bristol BS8 1TH, UK; s.j.lewis@bristol.ac.uk

**Keywords:** systematic review, pregnancy, childhood, mercury, methylmercury, neurodevelopment

## Abstract

Neurodevelopmental delays can interfere with children’s engagement with the world and further development, and may have negative consequences into adulthood. Mercury is highly toxic and may negatively influence neurodevelopment because it can freely cross the placenta and accumulate in the fetal brain. We searched four publication databases (Embase, PsycINFO, PubMed/MEDLINE, Scopus) for studies examining the relationship between early life mercury exposure and scores on neurodevelopmental performance measures in children aged 0 to 5 years old. Study quality was assessed using the National Institutes of Health (NIH) Quality Assessment Tool. Thirty-two prospective studies were included in the review. Neurodevelopmental performance was measured using 23 different scales, most commonly the Bayley Scales of Infant and Toddler Development (BSID). In most cases, the evidence for an association between mercury and neurodevelopment was weak. There did not appear to be exceptions for particular childhood ages, outcome scales, or mercury levels. The small number of results to the contrary were more likely to be studies which did not meet our high-quality criteria, and could be a consequence of multiple testing, selection bias, or incomplete confounder adjustment. Based on current evidence, dietary mercury exposure during pregnancy is unlikely to be a risk factor for low neurodevelopmental functioning in early childhood.

## 1. Introduction

During gestation and infancy, the brain undergoes rapid cell specialization and the shaping of neuronal networks which support neurodevelopmental functions such as cognitive, motor, language, and social skills [1]. Neurodevelopmental difficulties in early life may restrict a child’s range of experiences and lead to a cycle of impaired development that results in long-term harm to health and life opportunities [2,3]. For example, a child with motor difficulties may lose opportunities to play with peers, which then restricts the range of experiences and may affect social development. The prenatal environment is known to be a key factor in enabling optimal neurodevelopment [4], and exposure to toxic metals may play a role.

One such element is mercury (Hg). Direct contact with mercury (such as the inhalation of mercury vapors or skin contact) is extremely toxic and can result in organ failure and permanent damage throughout the central nervous system [5,6]. This is because mercury is highly reactive and disrupts cellular functions including protein and enzyme inhibition, epigenetic modifications, oxidative stress, and cellular death [7]. Mercury is deposited into all major oceans, primarily from industrial atmospheric emissions [8], where a cyclical process of methylation into organic Hg in the form of methylmercury (MeHg) occurs [9,10]. Aquatic MeHg can enter the human food chain following absorption by microorganisms and bioaccumulation into large fish such as tuna and swordfish which may be consumed by humans [8,11]. This dietary exposure is likely to increase in coming decades because of the decade-long time lag between atmospheric mercury emissions and ocean absorption, which means that that ocean Hg is expected to increase regardless of reductions in atmospheric emissions [8,12,13].

Methylmercury is the most common form of dietary Hg and is highly absorbable in the intestines [14,15], after which it can cross both the placenta [16] and blood-brain barrier [17,18]. Hg concentrations are higher in the neonatal umbilical cord than maternal blood [17,19], indicating that the fetus may be exposed to a significant proportion of the mother’s circulating mercury. Harmful concentrations may accumulate [20,21] during a critical window of neurological development [22], because the developing infant has both higher levels of mercury crossing the blood-brain barrier and less efficient clearance than adults [7,23,24].

Several epidemiological studies investigated prenatal mercury exposure and neurodevelopment. A review in 2018 [23] identified 11 studies which found evidence of neurodevelopmental harm from mercury exposure during pregnancy. A neurotoxicological review in the same year by Olivera et al. [7] concluded that the evidence from observational studies remained unclear. Neither of the reviews were systematic, and they did not evaluate study quality or compare study methodologies, which may have led to poor quality studies biasing their findings. A 2017 systematic review and meta-analysis of case-control studies compared mercury levels between individuals diagnosed with autism, a neurodevelopmental condition characterized by social communication impairments and repetitive behavior patterns, and typically developing individuals, and concluded that mercury causally increased the likelihood of autism [25]. However, this analysis did not consider potential reverse causation (e.g., autism sensory-seeking symptoms leading to higher mercury exposure), or which covariates were adjusted for in the analysis, an important omission given that there is the potential for confounding from factors such as fish intake [26]. Fish are a source of both methylmercury and of essential nutrients hypothesized to be involved in reducing the likelihood of autism such as long-chain polyunsaturated fatty acids (LCPUFA) [27,28].

This study aims to review the evidence concerning prenatal mercury exposure and early neurodevelopment systematically. Specific objectives are: (1) to identify systematically studies of mercury concentrations in maternal biomarkers and neurodevelopmental functioning in children aged 0 to 5 years old; (2) to assess the quality of studies; (3) to synthesize all results and identify the current pattern and strength of evidence.

## 2. Materials and Methods

### 2.1. Study Design and Protocol

This systematic review was designed to assess the evidence for an association between prenatal exposure to mercury and neurodevelopment in early childhood (0–5 years) from studies published up to December 2020. The age range of 0–5 years was selected because the first years of infancy are understood to form a critical window of brain development [29], and the clearance rate of mercury and methylmercury is such that prenatal exposure would not be expected to last beyond the first few years of life [30,31]. Studies which used biological samples to measure mercury concentrations during pregnancy or at delivery were included; ethylmercury was not included because the pathways to exposure and biological mechanisms are different from other forms of mercury. Studies were included which evaluated neurodevelopmental functioning using continuous scales (not neurodevelopmental diagnoses or symptom measures). The study design and protocol were registered with the International Prospective Register of Systematic Reviews (PROSPERO) on 23 November 2020, registration number CRD42020221146. Changes to the study and rationale are stated in Supplementary File Part 1.

### 2.2. Search Strategy

We identified search terms and subject headings (where possible) related to pregnancy, mercury, and neurodevelopment.

Search queries were developed for four bibliographic databases: Embase, MEDLINE (PubMed), PsycINFO, and Scopus. Animal studies were excluded, but there was no restriction by year of publication or language. Search queries are available in Supplementary File Part 2. We used Google search to identify relevant white papers, theses, and conference proceedings, using combinations of search terms and screening the first 5 pages of results. The reference list of each included paper was screened to identify additional studies missed by our database searches. The searches were run on 1 December 2020.

### 2.3. Study Selection

Search results were imported into Covidence systematic review software and automatically deduplicated [32]. Papers were screened in a two-stage process by K.D. and M.F. First, titles and abstracts were screened against our inclusion and exclusion criteria in Table 1. Second, papers which passed the first stage of screening were read in full, and a final decision was made by each reviewer. When studies were regarded as eligible by one reviewer but not the other, the studies were discussed to come to a consensus decision.

### 2.4. Data Extraction and Quality Assessment

The following data were extracted: Publication details, study type, location, mercury measurement method, neurodevelopmental functioning measurement tool, population, statistical methods, model adjustment variables, and results. A full list of data fields is shown in Supplementary File Part 3. The extracted data were checked by the second reviewer.

The quality of each study was assessed using the National Institutes of Health (NIH) Quality Assessment Tool [33], which was designed for prospective and cross-sectional studies (Supplementary File Part 4). The tool evaluates the design, sampling, measurement, and reporting quality of studies using 14 yes/no questions. Two further items were added which we judged important to consider: (1) whether studies selected covariates based on significance testing, which may encourage bias compared to selection based on prior evidence and theory [34]; (2) whether studies fully published the results of their planned analyses, or only those which met a significance threshold.

Studies were considered high quality if: (a) they met 12 or more of the 16 quality assessment criteria; and (b) they met item 14 of the NIH Quality Assessment Tool: *“Were key potential confounding variables measured and adjusted statistically for their impact on the relationship between exposure(s) and outcome(s)?”*. Item 14 was considered essential because the failure to adjust for important confounders would lead to bias in the model estimates. We identified (1) maternal socio-economic status or education, (2) fish or fatty acid intake, and (3) maternal smoking as key confounders [35,36,37,38,39,40,41].

### 2.5. Evidence Synthesis

We could not meta-analyze study results because of the numerous sources of heterogeneity in the design of studies, including mercury measurement, sample type, neurodevelopmental domain and assessment tool, the timing of exposure and outcome measurements, and type of statistical estimate. We instead synthesized results in a narrative review.

Studies used a variety of neurodevelopmental scales many of which measured different domains of neurodevelopmental functioning. We grouped the review into six broad domains which the neurodevelopmental outcomes could be mapped to:

1. Cognition and language as measured by the Bayley Scales of Infant and Toddler Development II (BSID-II) Mental Developmental Index (MDI).
2. Other measure of cognition, including attention, executive function, and memory.
3. Motor function, fine, and gross.
4. Communication and language development.
5. Social development.
6. General or composite measures of neurodevelopmental functioning.

## 3. Results

### 3.1. Study Characteristics

**Figure 1:**
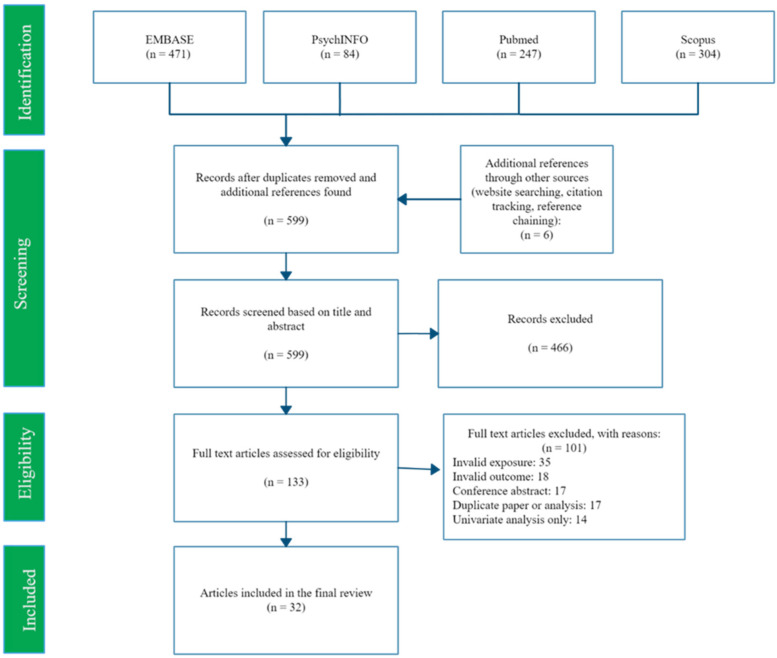
PRISMA flow diagram of search and selection process.

The search returned 593 potential studies after deduplication, and 6 more were found among reference lists. See Figure 1 for numbers at each stage of screening. We found 32 studies eligible for inclusion in this review, listed in Appendix A.

Neurodevelopmental functioning was measured using 23 scales, and the most common were variations of the BSID scale, found in 17 studies (Table 2). The BSID-II and Bayley-III) (also referred to as BSID-III) were designed to measure cognitive, motor, and language development in children aged 1 to 42 months. Scales are standardized across all ages to a mean of 100 and standard deviation of 15. The two versions are reviewed separately because performance on the two versions is correlated but not identical [42], and motor and language development is measured in a single scale in the second edition but not the third. Further scale details are available in Supplementary File Part 5.

All used a prospective design where tissue samples were taken prior to neurodevelopmental assessment, most frequently from the mother during pregnancy or delivery (Table 3). Tissue types commonly used to measure mercury concentrations were maternal whole blood (*n* = 10), hair (*n* = 10), or umbilical cord blood or tissue (*n* = 21). The studies took place in 16 countries where the primary source of mercury exposure was mostly fish or seafood consumption (e.g., [43]), but also from rice consumption [44], and proximity to artisanal tin [45] or gold mining [46].

Where reported, we recorded central tendency measures of mercury concentrations which are shown in Supplementary File Part 6. The mean Hg in maternal whole blood ranged from 0.64 [47] to 3.71 μg/L [48], and maternal hair sample concentrations from 0.3 [49] to 5.7 μg/g [50]. Not all umbilical cord blood or tissue concentrations were reported in comparable units, but one Taiwanese cohort had a mean cord blood Hg of 14.9 μg/L which was considerably higher than other studies [51].

Studies frequently did not meet NIH Quality Assessment criteria concerning transparency and reporting (results in Supplementary File Part 7). Most studies did not state their initial recruitment participation rate (item 3), and 5 did not explain why they recruited the reported sample size (item 5). Most studies either did not report their follow-up rate, or their loss to follow-up was greater than 20% of the initial population (item 13). Only 15 studies adjusted for all key confounders (item 14), and it was common for studies instead to select covariates using significance testing (*n* = 10, item 15). Overall, 15 studies passed our criteria for high quality.

### 3.2. Evidence Summary

This review contains 32 studies. Many of the studies included associations between mercury and both composite scores and subscales from neurodevelopmental assessment tools in their main results. It was common for studies to measure mercury concentrations in more than one tissue type and assess neurodevelopment at more than one time point (e.g., 12 months, 24 months). For these reasons there were many more results than studies: 175 estimates (median 4.5). Table 4, Table 5, Table 6, Table 7, Table 8 and Table 9 below include only studies which met our high-quality criteria, with results from all studies available in Supplementary File Part 8.

#### 3.2.1. Cognition and Language as Measured by the BSID-II MDI (*n* = 10)

The BSID-II is a standardized developmental test for infants and toddlers [52] and measures both cognitive and language development. It comprises the Psychomotor Development Index (PDI—reviewed later) and the MDI. We found 10 studies of maternal mercury Hg and childhood MDI, and the range of sample sizes was 87 to 1683. Whilst most observed negative associations, in almost all cases the confidence interval overlapped with the null and indicated only weak evidence of an association.

The pattern of results was consistent between studies using maternal whole blood, hair, and umbilical cord samples. Two South Korean studies using maternal whole blood found no strong evidence of an association in samples taken in both the first and third trimesters of pregnancy [48,53]. Similarly, weak evidence of negative associations was reported in studies of maternal hair mercury [50,54]. However, one study of MeHg exposure through rice consumption in China did identify stronger evidence albeit with wide confidence intervals (−4.9 MDI points per log μg/g, 95% CI: −9.7 to −0.1) [44].

Seven studies used umbilical cord blood or tissue [48,53,55,56,57,58,59]. One of those studies reported a negative association but did not adjust for fish or LCPUFA intake during pregnancy; the other studies did not find strong evidence of an association.

From the 10 studies of MDI, 4 were high quality [44,48,55,59]. Increased mercury exposure tended to be associated with lower MDI in these studies, but only one study reported an association which did not overlap with the null [44]. MDI represents both cognition and language development, but specific measures of cognition and language are reviewed in Section 2 and Section 4.

#### 3.2.2. Other Measure of Cognition, Including Attention, Executive Function, and Memory (*n* = 13)

The most common method used to measure cognition was the Bayley-III cognitive composite. This is different to the MDI of BSID-II because it is designed to measure cognitive performance more specifically, and includes the extreme ends of cognitive performance [60]. It was used in 7 studies of children in populations aged 12−40 months, none of which reported strong evidence of a negative association with maternal mercury concentrations [49,61,62,63,64,65,66]. This includes three studies which met our criteria for high quality, including an adjustment for key confounders [61,63,65].

Three studies used other measures of general cognition in cohorts that the authors hypothesized were exposed to high levels of mercury. Two cohorts of Japanese children at 42 months [67] and Taiwanese children aged 24 months [51] both suggested mercury exposure had occurred through seafood consumption, but neither reported strong evidence of an association between cord blood mercury concentrations and overall cognition. A US study of pregnant women in New York identified both seafood and air particles following the World Trade Center disaster as possible routes of exposure. It reported strong evidence of negative associations between umbilical cord mercury and full, performance, and verbal IQ scores in children aged 4 years old [58].

Memory [68], executive function [68], attention [47], visual matching [69], visual recognition [70], or object permanence and novelty fixation [55] were the subject of five studies of children at various stages of infancy up to 3 years old. In most cases, no strong evidence of an association was reported. However, performance on A-not-B tasks, which are intended to reflect object permanence, was negatively associated with cord Hg in one study [55]. Although confidence intervals overlapped with the null (−0.25 A-not-B 2 correct, 95% CI: −0.46 to 0.00; −0.22 A-not-B 3 correct, CI: −0.45 to 0.03; −0.21 perseverative errors score, CI: −0.53 to 0.09), this is likely because of small sample sizes in the three analyses (*n* = 73–79).

Overall, the 13 studies described in this section did not find strong evidence of an association. This finding was consistent in six studies identified as high quality [47,51,61,63,65,69]. A seventh high quality but small study (*n* = 73–79) also did not report strong evidence of an association, but as discussed in the previous paragraph appeared to be underpowered [55]. The source and level of mercury exposure varied but did not affect the pattern of results.

#### 3.2.3. Motor Function, Fine, and Gross (*n* = 23)

Ten studies used the BSID-II PDI to measure motor function in populations aged from 6 to 36 months [44,48,50,53,54,55,56,58,59,71]. The estimated association with maternal Hg was close to zero with no strong evidence of an association reported in five studies [53,54]—three of which were high quality [44,55,59]. Four other studies of maternal Hg reported 15 negative associations with PDI and 5 positive, but only 5 estimates—all negative—did not overlap with the null [48,50,56,58], and only one study was high quality [48]. While one further study reported evidence of a negative association (−0.119 PDI per Ln ng/g Hg, *p* = 0.009) with umbilical cord mercury concentrations and PDI [71], this study may be affected by reporting bias because it did not report all the results of its planned analyses.

Seven studies used the Bayley-III motor development scale, all including children of a roughly similar age (12 to 24 months) [49,61,63,64,65,66,72]. Maternal hair and umbilical cord sample mercury concentrations were in most cases found to be positively associated with overall motor development, but with confidence intervals overlapping the null in all studies [49,61,63,65,66]. The only strong evidence was a negative association reported between umbilical cord sample Hg and fine motor development [64], a finding not replicated in other studies of this sub-scale [61,63,72]. In studies using other measures of motor development, none reported strong evidence of an association with maternal mercury concentrations [46,47,51,68,69,73].

Of the six studies of motor development which reported evidence for a negative association, none adjusted for fish or fatty acid consumption. In the 11 studies which met our criteria for high quality, the pattern of evidence was different, with almost all results reporting no strong evidence of an association. In these high-quality studies, most appeared adequately powered based on their effect sizes and confidence intervals, but two which reported non-significant results had quite wide 95% confidence intervals which may indicate a lack of power [44,73]. Other factors such as study size or tissue sampled did not differ between studies marked high or not high quality.

In summary, there was little indication that mercury was associated with motor development. Unlike MDI or cognition where negative but non-significant point estimates were often found, for motor development there was no such pattern, and most estimates were close to zero.

#### 3.2.4. Communication and Language Development (*n* = 11)

Six studies using the Bayley-III language composite scale reported no strong evidence of an association at 12–24 months with maternal hair or umbilical cord mercury [49,61,63,64,65,66].

Two studies reported negative associations between maternal blood Hg and Peabody Picture Vocabulary performance [69] and The Malawi Developmental Assessment Tool language scale [46]. One of these met our high-quality criteria and measured second trimester erythrocyte concentrations, which primarily consist of MeHg [69]. Three other studies used umbilical cord samples, including one of 1054 children, and each reported no evidence of an association with language development [51,73,74].

In the six studies identified as high quality [61,63,65,69,73,74], most estimates were positive and overlapped the null. Low power did not appear to be an issue in most of these studies because estimates were close to the null without wide confidence intervals or relatively small sample sizes, although one study did appear to be underpowered (2.17 GDS language domain per Ln μg/L umbilical cord Hg, −1.88 to 6.21) [73].

#### 3.2.5. Social Development (*n* = 5)

Five studies measured social development in children aged between 6 and 18 months, three of which were high quality. No strong evidence of an association was found with maternal whole blood Hg [46,73], hair Hg [65], or umbilical cord samples [51,65,73,74]. Three studies were high quality [65,73,74]. One high-quality study contained wide confidence intervals which indicates possible low power; this study observed a positive effect (0.74 Gesell Developmental Schedules social domain score per Ln μg/L, −5.77 to 4.31, *n* = 410) [73].

#### 3.2.6. General or Composite Measures of Neurodevelopmental Functioning (*n* = 11)

Eleven studies used scales of neurodevelopment designed to assess overall functioning or could not otherwise be classified. Three of these were of neonatal functional ability and reflexes at 3 days old, two of which reported strong evidence of a negative association with mercury concentrations in maternal hair [43] and umbilical cord samples [66] taken at delivery. The third reported a positive association with umbilical cord Hg [75]. The two studies using umbilical cord samples both met 12 or more NIH quality criteria and adjusted for key confounders.

Six studies looked at mercury concentrations and general neurodevelopmental functioning during childhood [45,46,51,69,74,76]. One reported evidence of a negative association between maternal mercury and neurodevelopmental functioning [46]. However, this did not adjust for key confounding variables. Three studies met our high-quality criteria and reported either no evidence of an association [69,74] or strong evidence of a positive association in both fish eaters and non-fish eaters [76].

Two studies measured adaptive functioning, which encompasses the functional use of neurodevelopmental skills in daily life. One reported no strong evidence of an association [65], while the other found strong evidence of a positive association with umbilical cord Hg [73], which is the opposite direction to what would be expected if a neurotoxic effect was present. Both studies were high quality and adjusted for fish consumption. The latter suggested that despite adjustment for fish consumption, Hg levels may have been too low to overcome the benefits of fish intake (geometric mean of maternal blood Hg: 0.72 μg/L, IQR: 0.54 to 1.05 μg/L).

## 4. Discussion

This systematic review found 32 studies of childhood neurodevelopmental functioning in children aged from 3 days to 59 months old. The most widely used methods of measurement were the 2nd and 3rd versions of the BSID. Nineteen other measures were also used. Mercury concentrations were measured in tissues taken during pregnancy or at delivery.

The evidence for an association between mercury exposure and neurodevelopmental functioning was weak. While there were 17 results that provided evidence of a negative relationship, there were also 8 which indicated evidence of a positive association, and a further 150 where the estimated association overlapped with the null. We examined patterns of results by mercury biomarker, the timing of measurement, and child age, and the lack of evidence was consistent in all cases. Some authors suggested that whilst their findings were not indicative of a negative association, there may have been an age band where the neurological effects of mercury may be more clearly detectable. However, we did not identify stronger evidence at a particular age band. While three studies of neonatal behavior all reported evidence of an association [43,66,75] with maternal hair or umbilical cord mercury concentrations, the reliability of the neurological assessment of neonates may be less than that of older children. Neonates have a limited range of behaviors available, and there is a heightened role of the assessor in interpreting behavior, which may increase the risk of measurement error.

Fifteen high-quality studies of children aged 0 to 36 months were identified using a modified NIH QA tool. These studies were less likely to report strong evidence of an association, which may indicate where negative associations were reported; it may have been a consequence of study bias. While most studies appeared to be adequately powered, it is possible that five were underpowered because they reported wide confidence intervals which overlapped with the null [44,53,56,67,73].

The theorized adverse effects of mercury on neurodevelopment may be more detectable above a certain threshold of exposure. First, current measures of neurodevelopment may be unable to detect the small changes that occur at lower levels of exposure, which could still translate to significant adverse effects on a population level. Secondly, at lower levels there may adequate levels of nutrients which protect against oxidative stress [77] or aid mercury excretion [78]. This review included studies with mean mercury concentrations from 0.64 to 3.71 μg/L for whole blood samples, and 0.3 to 5.7 μg/g for maternal hair (Supplementary File Part 6). The findings of this review may not be applicable to populations with higher levels of mercury exposure, such as those that were reported in studies of the First Nations people in Canada [79] and the Faroe Islands [80].

There are few guidelines which consider a safe limit or reference value for circulating mercury during pregnancy and fetal health. The German FEA reported a reference value of 2.0 µg/L blood Hg for all adults [81]. The US EPA (2003) used data from three studies in the Faroe Islands, Seychelles Islands, and New Zealand to calculate a reference dose of blood Hg below which neurotoxicity is unlikely [82]. However, this estimate was based on multiple pharmacokinetic assumptions, and the studies themselves were not consistent in finding a threshold effect [83]. A US cohort not included in the EPA analysis estimated a reference dose level of 3.5 µg/L to avoid risks to the fetal nervous system [84]. Each of these estimates is towards the higher end of the distributions reported by studies in this review. The WHO suggested a safe limit for hair Hg of 10 ng/g [85] which is also above the mean from many studies in this review. Further guidelines exist in the context of fish consumption [86] which cannot easily be translated to circulating Hg concentrations. If neurotoxic effects from mercury are more detectable at higher levels of exposure, this does not appear to have been studied within the age range and other criteria included in our literature search.

Our findings do not replicate those of a systematic review of maternal dietary mercury intake which included 15 studies of neurodevelopmental outcomes in children up to 8 years old [87]. The review concluded there was clear evidence that maternal dietary exposure adversely affected childhood cognitive development, although this may be mitigated by nutrients in mercury-containing foods such as fish. Seven studies from this review were not included in our own because: they were carried out in children over 5 years of age, reported only univariate results, or duplicated an analysis contained in another included paper. This review included 17 studies which were not in the earlier review, most likely because the studies did not focus on dietary mercury exposure, and this may be a reason for the difference in our conclusions. A systematic review of fish consumption during pregnancy and childhood neurodevelopment concluded there was moderate evidence of a beneficial effect [88]. Our finding that there is a lack of evidence for a negative association between mercury concentrations and early childhood neurodevelopment is consistent with this, but not directly comparable.

There are several limitations to our review. First, it includes studies using a wide variety of neurodevelopmental measures, which measure different domains of neurodevelopmental functioning, and some measures are not directly comparable. To address this, we grouped and summarized results by the aspect of neurodevelopment being measured, such as cognition or motor function. However, these groupings are approximate generalizations, and there may be alternative methods of summarizing the results which more accurately represent the underlying neurodevelopmental dimensions. Additionally, we did not appraise the ability of the scales to represent neurodevelopmental functioning accurately in young children, and it is unlikely that all measures used in our included studies are equally valid. Finally, this review did not include studies which measured neurodevelopmental condition diagnoses or symptoms. It may be that different results are seen in those outcomes.

The second limitation of the review is the assumption that mercury concentrations in maternal whole blood, hair, placenta, and umbilical cord samples are correlated with prenatal mercury exposure. While the intercorrelation between these biomarkers is well established (e.g., [89,90]), there is limited evidence to confirm the hypothesized correlation with mercury concentrations in the fetus itself [16]. It may be that maternal blood or hair mercury correlates poorly with fetal mercury exposure, which would affect the inclusion criteria of this review. Thirdly, the high degree of heterogeneity in the methods of included studies meant it was not possible to synthesize results in a quantitative manner such as through meta-analysis. Finally, it is possible that despite finding limited evidence of an association between Hg and neurodevelopment, many studies included in the review were small and underpowered to detect more modest effects. Therefore, there may still be a small adverse relationship which would translate to an important deficit in, for example, intellectual capital, in a large population.

This review’s strengths are first that we conducted a literature search on a wide range of neurodevelopmental functions in infants and young children, and were able to find a large number of studies. This enabled us to compare the pattern of results along many study dimensions such as the characteristics of study populations and the exposure and outcome measurement timing and methods. Our findings were consistent across these study characteristics, but there were indications of heterogeneity. Some studies reported positive associations while others were negative, and there were a minority of studies reporting strong evidence of an association in both directions. Secondly, the review was conducted in a systematic way, with the results and conclusions based on all the reported results in the included studies. This may give a more accurate and comprehensive overview of the evidence compared to previous narrative reviews which tended to discuss only significant results. Third, we conducted the review in a transparent manner by following a registered protocol and publishing all data used to inform the review. Finally, we identified high-quality studies using an externally developed quality assessment tool and by examining the specific model parameterization used in each study.

## 5. Conclusions

At the levels of mercury recorded in studies included in this review, the evidence for an association between prenatal mercury concentrations and neurodevelopmental functioning in children from 0 to 5 years is weak. No pattern was identified by the age of child or study methodology. Any adverse effect may also be too small to be clinically detectable. Fish contains many essential nutrients involved in brain development such as LCPUFA, so where fish is the main source of dietary Hg, these other nutrients may compensate against the toxic effects of mercury. Future studies may wish to focus on populations with higher levels of mercury exposure or consider alternative study designs with different assumptions and limitations, such as natural experiments or genetic analyses.

## Figures and Tables

**Table 1:** Criteria for including or excluding papers from this systematic review.

Include	Exclude
1. Study of total mercury, inorganic, organic, or methylmercury compounds.	1. Studies other compounds including ethylmercury.
2. Measures mercury in pregnant women.	2. Measures mercury in other populations.
3. Measures mercury concentrations in biological samples: blood (whole, erythrocyte, plasma, serum), urine, cord blood/tissue, placenta, or hair.	3. Uses any other measure of mercury exposure.
4. Measures neurodevelopmental functioning (i.e.: cognition, attention, memory, intelligence, fine/gross motor development, receptive/expressive language ability, communicative ability, social development, or overall neurodevelopment) in children aged 0 to 5 years old.	4. Measures neurodevelopmental functioning in older children or adults, or only measures diagnoses or symptoms of neurodevelopmental disorders.
5. Reports association between mercury and neurodevelopmental functioning	5. Does not report associations between mercury and specified outcomes.
6. Study reports results from multivariable analysis methods.	6. Study reports results only from univariable methods such as correlations or *t*-tests.

**Table 2:** Measures of neurodevelopmental functioning in the included studies.

Abbreviation	Name	*n*
A-not-B	A-not-B test	1
BSID-II	Bayley Scales of Infant and Toddler Development—Second Edition	9
Bayley-III/BSID-III	Bayley Scales of Infant and Toddler Development—Third Edition	8
CDI	MacArthur-Bates Communicative Development Inventories	1
CDIIT	Comprehensive Developmental Inventory for Infants and Toddlers	1
DDST	Denver Developmental Screening Test	1
DDST (modified)	Modified version of the Denver Developmental Screening Test	1
FTII	Fagan Test of Infant Intelligence	1
GDS	Gesell developmental schedules	2
K-ABC	Kaufman Assessment Battery for Children	1
K-BSID-II	Korean adapted version of Bayley Scales of Infant and Toddler Development II	1
KSPD	Kyoto Scale of Psychological Development	1
MCDI	MacArthur Communicative Development Inventory	1
MDAT	Malawi Developmental Assessment Tool	1
MSCA	McCarthy Scales of Children’s Abilities	1
NBAS	Neonatal Behavioral Assessment Scale	1
NBNA	Neonatal Behavioral Neurological Assessment	2
NNNS	NICU Network Neurobehavioral Scale	1
PPVT	Peabody Picture Vocabulary Test	1
SMS	Social Maturity Scale (Vineland)	1
VRM	Visual recognition memory	1
WPPSI-R	Wechsler Preschool and Primary Scales of Intelligence-Revised	2
WRAVMA	Wide Range Assessment of Visual Motor Abilities	1

**Table 3:** Study characteristics.

Country	*n* ^1^		*n* ^1^
Brazil	1	Republic of Seychelles	2
Canada	1	Slovenia	2
China	4	South Korea	3
Croatia	4	Spain	2
Greece	1	Taiwan	1
Italy	4	Tanzania	1
Japan	3	United Kingdom	2
Poland	2	USA	4
Mercury source (all maternal)	*n*	Mercury analysis	*n*
Whole blood	10	Atomic absorption spectroscopy (AAS)	3
Erythrocyte	1	Cold vapour atomic absorption spectrometry (CVAAS)	22
Hair	10	Inductively coupled plasma mass spectrometry (ICP-DRC-MS)	1
Placenta	1	Inductively coupled plasma mass spectrometry (ICP-MS)	4
Umbilical cord	21		

^1^ Studies may be counted more than once if they include more than one country, mercury source, or mercury analysis method.

**Table 4:** Results from studies identified as high quality which measured cognition and language in the BSID:II MDI scale (*n* = 4).

Study	*n*	Exposure	Units	Outcome	Time of Outcome	Estimate	95% CI	*p*-Value
Boucher et al. (2014)	87	Umbilical cord	Ln μg/L	BSID-II: MDI	11 months	0.08	−0.15 to 0.33	
Kim Y et al. (2018)	595	Umbilical cord	μg/L	BSID-II: MDI	12 months	−0.04	−0.39 to 0.32	0.85
Kim Y et al. (2018)	523	Umbilical cord	μg/L	BSID-II: MDI	24 months	−0.03	−0.36 to 0.3	0.87
Kim Y et al. (2018)	438	Umbilical cord	μg/L	BSID-II: MDI	36 months	0.17	−0.26 to 0.6	0.43
Kim Y et al. (2018)	662	Umbilical cord	μg/L	BSID-II: MDI	6 months	−0.03	−0.27 to 0.21	0.8
Kim Y et al. (2018)	763	Whole blood (early pregnancy)	μg/L	BSID-II: MDI	12 months	−0.32	−0.89 to 0.26	0.28
Kim Y et al. (2018)	614	Whole blood (late pregnancy)	μg/L	BSID-II: MDI	12 months	−0.07	−0.69 to 0.55	0.82
Kim Y et al. (2018)	686	Whole blood (early pregnancy)	μg/L	BSID-II: MDI	24 months	−0.06	−0.63 to 0.51	0.83
Kim Y et al. (2018)	564	Whole blood (late pregnancy)	μg/L	BSID-II: MDI	24 months	−0.46	−1.03 to 0.12	0.12
Kim Y et al. (2018)	557	Whole blood (early pregnancy)	μg/L	BSID-II: MDI	36 months	−0.28	−0.89 to 0.32	0.36
Kim Y et al. (2018)	460	Whole blood (late pregnancy)	μg/L	BSID-II: MDI	36 months	−0.25	−0.88 to 0.38	0.43
Kim Y et al. (2018)	847	Whole blood (early pregnancy)	μg/L	BSID-II: MDI	6 months	−0.41	−0.81 to −0.003	0.048
Kim Y et al. (2018)	689	Whole blood (late pregnancy)	μg/L	BSID-II: MDI	6 months	−0.13	−0.56 to 0.29	0.54
Llop et al. (2012)	1683	Umbilical cord	Ln µg/L	BSID-II: MDI	14 months	0.16	−0.12 to 0.45	
Rothenberg et al. (2016)	270	Hair	Ln μg/g	BSID-II: MDI	12 months	−4.9	−9.7 to −0.1	

**Table 5:** Results from studies identified as high quality which measured cognition (*n* = 7).

Study	*n*	Exposure	Units	Outcome	Time of Outcome	Estimate	95% CI	*p*-Value
Barbone et al. (2019)	1083	Hair	ng/g	Bayley-III: Cognitive composite	18 months	0.2	−0.29 to 0.69	
Barbone et al. (2019)	829	Umbilical cord	ng/g	Bayley-III: Cognitive composite	18 months	0.13	−0.39 to 0.64	
Barbone et al. (2019)	636	Whole blood	ng/g	Bayley-III: Cognitive composite	18 months	−0.09	−0.61 to 0.43	
Boucher et al. (2014)	77	Umbilical cord	ln μg/L	A-not-B: 2 correct	11 months	−0.25	−0.46 to 0.00	
Boucher et al. (2014)	77	Umbilical cord	ln μg/L	A-not-B: 3 correct	11 months	−0.22	−0.45 to 0.03	
Boucher et al. (2014)	73	Umbilical cord	ln μg/L	Perseverative errors	11 months	−0.21	−0.53 to 0.09	
Boucher et al. (2014)	89	Umbilical cord	ln μg/L	FTII: Novelty preference	6.5 months	0.0	−0.19 to 0.19	
Boucher et al. (2014)	89	Umbilical cord	ln μg/L	FTIII: Fixation duration	6.5 months	0.13	−0.03 to 0.29	
Lin et al. (2013)	230	Umbilical cord	Hg ≥ 19.78 μg/L	CDIIT: Cognitive	24 months	0.09		NS
Nisevic et al. (2019)	257	Umbilical cord	μg/L	Bayley-III: Cognitive composite	18 months	0.14		0.34
Oken et al. (2008)	341	Erythrocyte	ng/g	WRAVMA matching	36 months	−0.2	−0.6 to 0.2	
Valent et al. (2013)	505	Hair	Ln ng/g	Bayley-III: Cognitive composite	18 months	−0.002		0.99
Valent et al. (2013)	378	Umbilical cord	Ln ng/g	Bayley-III: Cognitive composite	18 months	0.05		0.92
Xu et al. (2016)	270	Umbilical cord	μg/L	NNNS: Attention	5 weeks	0.12		0.23
Xu et al. (2016)	344	Whole blood	μg/L	NNNS: Attention	5 weeks	0.15		0.22

**Table 6:** Results from studies identified as high quality which measured motor function (*n* = 11).

Study	*n*	Exposure	Units	Outcome	Time of Outcome	Estimate	95% CI	*p*-Value
Barbone et al. (2019)	1082	Hair	ng/g	Bayley-III: Fine Motor scale	18 months	−0.03	−0.11 to 0.06	
Barbone et al. (2019)	1081	Hair	ng/g	Bayley-III: Gross Motor scale	18 months	−0.01	−0.07 to 0.05	
Barbone et al. (2019)	1083	Hair	ng/g	Bayley-III: Motor development	18 months	−0.12	−0.47 to 0.22	
Barbone et al. (2019)	635	Umbilical cord	ng/g	Bayley-III: Gross Motor scale	18 months	−0.02	−0.08 to 0.05	
Barbone et al. (2019)	892	Umbilical cord	ng/g	Bayley-III: Motor development	18 months	−0.11	−0.47 to 0.25	
Barbone et al. (2019)	636	Whole blood	ng/g	Bayley-III: Fine Motor scale	18 months	0.05	−0.04 to 0.15	
Barbone et al. (2019)	890	Whole blood	ng/g	Bayley-III: Gross Motor scale	18 months	−0.03	−0.09 to 0.03	
Barbone et al. (2019)	636	Whole blood	ng/g	Bayley-III: Motor development	18 months	0.11	−0.25 to 0.48	
Boucher et al. (2014)	87	Umbilical cord	ln μg/L	BSID-II: PDI	11 months	0.01	−0.24 to 0.25	
Hu et al. (2016)	410	Umbilical cord	Ln μg/L	GDS: Fine motor domain	12 months	−2.62	−7.78 to 2.55	
Hu et al. (2016)	410	Umbilical cord	Ln μg/L	GDS: Gross motor domain	12 months	1.95	−3.08 to 6.98	
Hu et al. (2016)	410	Whole blood	Ln μg/L	GDS: Fine motor domain	12 months	2.69	−3.37 to 8.74	
Hu et al. (2016)	410	Whole blood	Ln μg/L	GDS: Gross motor domain	12 months	3.26	−2.72 to 9.24	
Kim Y et al. (2018)	595	Umbilical cord	μg/L	BSID-II: PDI	12 months	0.14	−0.23 to 0.52	0.45
Kim Y et al. (2018)	523	Umbilical cord	μg/L	BSID-II: PDI	24 months	0.16	−0.17 to 0.48	0.34
Kim Y et al. (2018)	438	Umbilical cord	μg/L	BSID-II: PDI	36 months	−0.13	−0.55 to 0.27	0.53
Kim Y et al. (2018)	662	Umbilical cord	μg/L	BSID-II: PDI	6 months	−0.2	−0.45 to 0.15	0.33
Kim Y et al. (2018)	763	Whole blood (early pregnancy)	μg/L	BSID-II: PDI	12 months	0.3	−0.31 to 0.91	0.34
Kim Y et al. (2018)	614	Whole blood (late pregnancy)	μg/L	BSID-II: PDI	12 months	0.27	−0.38 to 0.93	0.41
Kim Y et al. (2018)	686	Whole blood (early pregnancy)	μg/L	BSID-II: PDI	24 months	−0.17	−0.74 to 0.4	0.56
Kim Y et al. (2018)	564	Whole blood (late pregnancy)	μg/L	BSID-II: PDI	24 months	−0.09	−0.67 to 0.48	0.75
Kim Y et al. (2018)	557	Whole blood (early pregnancy)	μg/L	BSID-II: PDI	36 months	−0.11	−0.7 to 0.47	0.70
Kim Y et al. (2018)	460	Whole blood (late pregnancy)	μg/L	BSID-II: PDI	36 months	−0.58	−1.19 to 0.03	0.06
Kim Y et al. (2018)	847	Whole blood (early pregnancy)	μg/L	BSID-II: PDI	6 months	−0.55	−1.05 to −0.05	0.03
Kim Y et al. (2018)	689	Whole blood (late pregnancy)	μg/L	BSID-II: PDI	6 months	−0.27	−0.78 to 0.25	0.31
Llop et al. (2012)	1683	Umbilical cord	Ln µg/L	BSID-II: PDI	14 months	−0.05	−0.79 to 0.68	
Nisevic et al. (2019)	257	Umbilical cord	μg/L	Bayley-III: Fine Motor scale	18 months	−0.07		0.78
Nisevic et al. (2019)	257	Umbilical cord	μg/L	Bayley-III: Gross Motor scale	18 months	0.08		0.71
Nisevic et al. (2019)	257	Umbilical cord	μg/L	Bayley-III: Motor development	18 months	0.01		0.92
Oken et al. (2008)	341	Erythrocyte	ng/g	WRAVMA drawing	36 months	0.1	−0.2 to 0.4	
Oken et al. (2008)	341	Erythrocyte	ng/g	WRAVMA pegboard	36 months	0.03	−0.3 to 0.3	
Rothenberg et al. (2016)	270	Hair	Ln μg/g	BSID-II: PDI	12 months	−2.7	−8.3 to 2.9	
Tatsuta et al. (2017)	566	Umbilical cord	Ln ng/g	BSID-II: PDI	18 months	−0.12		0.009
Valent et al. (2013)	505	Hair	Ln ng/g	Bayley-III: Motor development	18 months	−0.19		0.62
Valent et al. (2013)	378	Umbilical cord	Ln ng/g	Bayley-III: Motor development	18 months	0.16		0.68
Xu et al. (2016)	270	Umbilical cord	μg/L	NNNS: Asymmetry (Male)	5 weeks	0.1		0.36
Xu et al. (2016)	270	Umbilical cord	μg/L	NNNS: Asymmetry (Female)	5 weeks	0.07		0.4
Xu et al. (2016)	270	Umbilical cord	μg/L	NNNS: Handling	5 weeks	−0.02		0.35
Xu et al. (2016)	344	Whole blood	μg/L	NNNS: Asymmetry (Male)	5 weeks	−0.13		0.3
Xu et al. (2016)	344	Whole blood (Female)	μg/L	NNNS: Asymmetry (Female)	5 weeks	0.08		0.43
Xu et al. (2016)	344	Whole blood	μg/L	NNNS: Handling	5 weeks	−0.001		0.98

**Table 7:** Results from studies identified as high quality which measured communication and language development (*n* = 6).

Study	*n*	Exposure	Units	Outcome	Time of Outcome	Estimate	95% CI	*p*-Value
Barbone et al. (2019)	1272	Hair	ng/g	Bayley-III: Expressive Communication scale	18 months	0.04	−0.06 to 0.13	
Barbone et al. (2019)	1086	Hair	ng/g	Bayley-III: Language composite	18 months	0.55	0.05 to 1.05	
Barbone et al. (2019)	1075	Hair	ng/g	Bayley-III: Receptive Communication scale	18 months	0.12	0.02 to 0.22	
Barbone et al. (2019)	1070	Umbilical cord	ng/g	Bayley-III: Expressive Communication scale	18 months	0.01	−0.09 to 0.11	
Barbone et al. (2019)	896	Umbilical cord	ng/g	Bayley-III: Language composite	18 months	0.25	−0.29 to 0.78	
Barbone et al. (2019)	887	Umbilical cord	ng/g	Bayley-III: Receptive Communication scale	18 months	0.12	−0.08 to 0.32	
Barbone et al. (2019)	727	Whole blood	ng/g	Bayley-III: Expressive Communication scale	18 months	0.13	−0.22 to 0.48	
Barbone et al. (2019)	628	Whole blood	ng/g	Bayley-III: Receptive Communication scale	18 months	−0.02	−0.12 to 0.08	
Daniels et al. (2004)	1054	Umbilical cord	μg/g	MCDI: Vocabulary Comprehension	15 months	6.1		0.8
Daniels et al. (2004)	1054	Umbilical cord	μg/g	DDST: Language	18 months	0.1		0.9
Hu et al. (2016)	410	Umbilical cord	Ln μg/L	GDS: Language domain	12 months	2.17	−1.88 to 6.21	
Hu et al. (2016)	410	Whole blood	Ln μg/L	GDS: Language domain	12 months	1.92	−3.61 to 7.46	
Nisevic et al. (2019)	257	Umbilical cord	μg/L	Bayley-III: Language composite	18 months	−0.05		0.74
Oken et al. (2008)	341	Erythrocyte	ng/g	PPVT	36 months	−0.4	−0.8 to −0.1	
Valent et al. (2013)	505	Hair	Ln ng/g	Bayley-III: Language composite	18 months	0.85		0.11
Valent et al. (2013)	378	Umbilical cord	Ln ng/g	Bayley-III: Language composite	18 months	0.41		0.46

**Table 8:** Results from studies identified as high quality which measured social development (*n* = 3).

Study	*n*	Exposure	Units	Outcome	Time of Outcome	Estimate	95% CI	*p*-Value
Daniels et al. (2004)	1054	Umbilical cord	μg/g	MCDI: Social activity	15 months	−0.2		0.9
Daniels et al. (2004)	1054	Umbilical cord	μg/g	DDST: Social Activity	18 months	0.5		0.8
Hu et al. (2016)	410	Umbilical cord	Ln μg/L	GDS: Social domain	12 months	4.06	0.51 to 7.62	
Hu et al. (2016)	410	Whole blood	Ln μg/L	GDS: Social domain	12 months	0.74	−5.77 to 4.31	
Valent et al. (2013)	505	Hair	Ln ng/g	Bayley-III: Social-emotional	18 months	1.77		0.11
Valent et al. (2013)	378	Umbilical cord	Ln ng/g	Bayley-III: Social-emotional	18 months	−0.07		0.95

**Table 9:** Results from studies identified as high quality which measured general or composite measures of neurodevelopmental functioning (*n* = 6).

Study	*n*	Exposure	Units	Outcome	Time of Outcome	Estimate	95% CI	*p*-Value
Daniels et al. (2004)	1054	Umbilical cord	μg/g	DDST: Total	18 months	0.4		0.9
Golding et al. (2016)	2643	Whole blood	μg/L	DDST-II	18 months	0.49	0.1 to 0.88	0.01
Golding et al. (2016)	2452	Whole blood	μg/L	DDST-II	30 months	0.23	−0.08 to 0.53	0.15
Golding et al. (2016)	2394	Whole blood	μg/L	DDST-II	32 months	0.43	0.08 to 0.78	0.02
Golding et al. (2016)	2721	Whole blood	μg/L	DDST-II	6 months	0.51	0.05 to 1	0.03
Hu et al. (2016)	410	Umbilical cord	Ln μg/L	GDS: Adaptive domain	12 months	4.22	0.77 to 7.67	
Hu et al. (2016)	410	Whole blood	Ln μg/L	GDS: Adaptive domain	12 months	0.65	−4.3 to 5.59	
Oken et al. (2008)	341	Erythrocyte	ng/g	WRAVMA total	36 months	−0.06	−0.4 to 0.2	
Suzuki et al. (2010)	498	Hair	μg/g	NBAS	3 days	−0.12		<0.05
Valent et al. (2013)	362	Hair	Ln ng/g	Bayley-III: Adaptive behaviour	18 months	0.55		0.56
Valent et al. (2013)	271	Umbilical cord	Ln ng/g	Bayley-III: Adaptive behaviour	18 months	−0.57	−0.15 to 0.33	0.57

## Data Availability

All extracted data used to write this review are included in Appendix A and Supplementary File Parts 1–8.

## Supplementary Materials

The following are available online at https://www.mdpi.com/article/10.3390/ijerph19041976/s1, Part 1: Changes to the protocol, Part 2: Search terms, Part 3: fields used in data extraction, Part 4: Quality assessment tool, Part 5: Measures of neurodevelopmental functioning in the included studies, Part 6: Mercury exposure characteristics in included studies, Part 7: Results from NIH Quality Assessment, Part 8: Results from all studies.

## Appendix A. Included Studies

**Table:**

**Study** **(Year)**	**Reference**	**Country**	**Study Design**	**Exposure**	**Timing of** **Exposure**	**Outcome ^1^**	**Timing of Outcome**	**Sample Size ^2^**	**High Quality ^3^**
Barbone et al. (2019)	[61]	Italy, Slovenia, Croatia, Greece	Prospective	Hair Whole blood Umbilical cord	20 weeks—delivery 20 weeks—delivery At delivery	Bayley-III	18 months	628–1086	Yes
Boucher et al. (2014)	[55]	Canada	Prospective	Umbilical cord	At delivery	FTII, A-not-B test, BSID-II	6.5, 11 months	94	Yes
Castriotta et al. (2020)	[62]	Italy	Prospective	Umbilical cord	20–32 weeks gestation	Bayley-III cognitive	40 months	323	No
Daniels et al. (2004)	[74]	United Kingdom	Prospective	Umbilical cord	At delivery	MCDI, DDST	15, 18 months	1054	Yes
Davidson et al. (2008)	[50]	Republic of Seychelles	Prospective	Hair	At delivery	BSID-II	9, 30 months	225–228	No
Freire et al. (2018)	[68]	Spain	Prospective	Placenta	At delivery	MSCA	48-60 months	302	No
Golding et al. (2016)	[76]	United Kingdom	Prospective	Whole blood	11 weeks gestation	Modified DDST	6, 18, 30, 42 months	2394–2721	No
Hu et al. (2016)	[73]	China	Prospective	Whole blood Umbilical cord	At delivery	GDS	12 months	410	Yes
Jedrychowski et al. (2007)	[56]	Poland	Prospective	Umbilical cord	At delivery	BSID-II	12, 24, 36 months	270–374	No
Kim S et al. (2008)	[57]	South Korea	Prospective	Whole blood Umbilical cord	At delivery	BSID-II, SMS	13-24 months	49–118	No
Kim Y et al. (2018)	[48]	South Korea	Prospective	Whole blood Whole blood Umbilical cord	12–20 weeks gestation 28–42 weeks gestation At delivery	BSID-II	6, 12, 24, 36 months	414–790	Yes
Lederman et al. (2008)	[58]	USA	Prospective	Umbilical cord	At delivery	BSID-II, WPPSI-R	12, 24, 36, 48 months	107-132	No
Lin et al. (2013)		Taiwan	Prospective	Umbilical cord	At delivery	CDIIT	24 months	230	No
Llop et al. (2012)	[59]	Spain	Prospective	Umbilical cord	At delivery	BSID-II	14 months	1683	Yes
Marques et al. (2009)	[45]	Brazil	Prospective	Hair	At delivery	GDS	6, 36, 60 months	82	No
Nisevic et al. (2019)	[63]	Croatia, Italy	Prospective	Umbilical cord	At delivery	Bayley-III	18 months	257	Yes
Nyanza et al. (2020)	[46]	Tanzania	Prospective	Whole blood	16–27 weeks gestation	MDAT	6–12 months	429	No
Oken et al. (2005)	[70]	USA	Prospective	Hair	At delivery	VRM	6 months	135	No
Oken et al. (2008)	[69]	USA	Prospective	Erythrocyte	2nd trimester	PPVT, WRAVMA	36 months	341	Yes
Polanska et al. (2013)	[49]	Poland	Prospective	Hair	30–34 weeks gestation	Bayley-III	12–24 months	303	No
Prpic et al. (2017)	[72]	Croatia	Prospective	Umbilical cord	At delivery	Bayley-III	18 months	135	Yes
Rothenberg et al. (2016)	[44]	China	Prospective	Whole blood Hair	At delivery	BSID-II	12 months	270	Yes
Shah-Kulkarni et al. (2020)	[53]	South Korea	Prospective	Whole blood Whole blood Umbilical cord	12–20 weeks gestation > 28 weeks gestation At delivery	K-BSID-II	6 months	321-467	No
SnojTratnik et al. (2017)	[64]	Slovenia, Croatia	Prospective	Hair Umbilical cord	34 weeks gestation/at delivery At delivery	Bayley-III	16–20 months	280–357	No
Strain et al. (2015)	[54]	Republic of Seychelles	Prospective	Hair	At delivery	BSID-II, CDI	20 months	1241–1265	No
Suzuki et al. (2010)	[43]	Japan	Prospective	Hair	At delivery	NBAS	3 days	498	Yes
Tatsuta et al. (2014)	[67]	Japan	Prospective	Umbilical cord	At delivery	K-ABC	42 months	287	No
Tatsuta et al. (2017)	[71]	Japan	Prospective	Umbilical cord	At delivery	BSID-II, KSPD	18 months	566	Yes
Valent et al. (2013)	[65]	Italy	Prospective	Hair Umbilical cord	20–22 weeks gestationAt delivery	Bayley-III	18 months	271–505	Yes
Wang et al. (2019)	[66]	China	Prospective	Umbilical cord	At delivery	NBNA Bayley-III	3 days18 months	172–265	No
Wu et al. (2014)	[75]	China	Prospective	Umbilical cord	At delivery	NBNA	3 days	418	No
Xu et al. (2016)	[47]	USA	Prospective	Whole blood Umbilical cord	16 weeks—delivery At delivery	NNNS	5 weeks	344	Yes

^1^ For explanation of abbreviations, see Table 2. ^2^ Size of primary analysis, or a range if there were multiple primary analyses. ^3^ 12 or more quality assessment criteria met, including adjustment for key confounders.

## References

[1] Tierney A.L., Nelson C.A. (2009). Brain Development and the Role of Experience in the Early Years. Zero Three.

[2] Cusick S.E., Georgieff M.K. (2016). The Role of Nutrition in Brain Development: The Golden Opportunity of the “First 1000 Days”. J. Pediatr..

[3] Walker S.P., Wachs T.D., Meeks Gardner J., Lozoff B., Wasserman G.A., Pollitt E., Carter J.A. (2007). Child development: Risk factors for adverse outcomes in developing countries. Lancet.

[4] Fitzgerald E., Hor K., Drake A.J. (2020). Maternal influences on fetal brain development: The role of nutrition, infection and stress, and the potential for intergenerational consequences. Early Hum. Dev..

[5] Bernhoft R.A. (2012). Mercury toxicity and treatment: A review of the literature. J. Environ. Public Health.

[6] Syversen T., Kaur P. (2012). The toxicology of mercury and its compounds. J. Trace Elem. Med. Biol..

[7] Oliveira C.S., Nogara P.A., Ardisson-Araújo D.M.P., Aschner M., Rocha J.B.T., Dórea J.G. (2018). Neurodevelopmental Effects of Mercury. Adv. Neurotoxicol..

[8] Mason R.P., Choi A.L., Fitzgerald W.F., Hammerschmidt C.R., Lamborg C.H., Soerensen A.L., Sunderland E.M. (2012). Mercury biogeochemical cycling in the ocean and policy implications. Environ. Res..

[9] Lin H., Ascher D.B., Myung Y., Lamborg C.H., Hallam S.J., Gionfriddo C.M., Holt K.E., Moreau J.W. (2021). Mercury methylation by metabolically versatile and cosmopolitan marine bacteria. ISME J..

[10] Munson K.M., Lamborg C.H., Boiteau R.M., Saito M.A. (2018). Dynamic mercury methylation and demethylation in oligotrophic marine water. Biogeosciences.

[11] Balshaw S., Edwards J., Daughtry B., Ross K. (2007). Mercury in seafood: Mechanisms of accumulation and consequences for consumer health. Rev. Environ. Health.

[12] United Nations Environment Programme (2019). Global Mercury Assessment 2018.

[13] Sunderland E.M., Krabbenhoft D.P., Moreau J.W., Strode S.A., Landing W.M. (2009). Mercury sources, distribution, and bioavailability in the North Pacific Ocean: Insights from data and models. Glob. Biogeochem. Cycles.

[14] Hong Y.-S., Kim Y.-M., Lee K.-E. (2012). Methylmercury exposure and health effects. J. Prev. Med. Public Health.

[15] Bradley M.A., Barst B.D., Basu N. (2017). A Review of Mercury Bioavailability in Humans and Fish. Int. J. Environ. Res. Public Health.

[16] Tong M., Yu J., Liu M., Li Z., Wang L., Yin C., Ren A., Chen L., Jin L. (2021). Total mercury concentration in placental tissue, a good biomarker of prenatal mercury exposure, is associated with risk for neural tube defects in offspring. Environ. Int..

[17] Chen Z., Myers R., Wei T., Bind E., Kassim P., Wang G., Ji Y., Hong X., Caruso D., Bartell T. (2014). Placental transfer and concentrations of cadmium, mercury, lead, and selenium in mothers, newborns, and young children. J. Expo. Sci. Environ. Epidemiol..

[18] Aschner M., Aschner J.L. (1990). Mercury neurotoxicity: Mechanisms of blood-brain barrier transport. Neurosci. Biobehav. Rev..

[19] Sakamoto M., Chan H.M., Domingo J.L., Koriyama C., Murata K. (2018). Placental transfer and levels of mercury, selenium, vitamin E, and docosahexaenoic acid in maternal and umbilical cord blood. Environ. Int..

[20] Aberg B., Ekman L., Falk R., Greitz U., Persson G., Snihs J.O. (1969). Metabolism of Methyl Mercury (^203^Hg) Compounds in Man. Arch. Environ. Health Int. J..

[21] Jo S., Woo H.D., Kwon H.-J., Oh S.-Y., Park J.-D., Hong Y.-S., Pyo H., Park K.S., Ha M., Kim H. (2015). Estimation of the Biological Half-Life of Methylmercury Using a Population Toxicokinetic Model. Int. J. Environ. Res. Public Health.

[22] Vohr B.R., Poggi Davis E., Wanke C.A., Krebs N.F. (2017). Neurodevelopment: The Impact of Nutrition and Inflammation During Preconception and Pregnancy in Low-Resource Settings. Pediatrics.

[23] Bellinger D.C. (2018). Environmental chemical exposures and neurodevelopmental impairments in children. Pediatr. Med..

[24] Rand M.D., Caito S.W. (2019). Variation in the biological half-life of methylmercury in humans: Methods, measurements and meaning. Biochim. Biophys. Acta (BBA) Gen. Subj..

[25] Jafari T., Rostampour N., Fallah A.A., Hesami A. (2017). The association between mercury levels and autism spectrum disorders: A systematic review and meta-analysis. J. Trace Elem. Med. Biol..

[26] Choi A.L., Cordier S., Weihe P., Grandjean P. (2008). Negative confounding in the evaluation of toxicity: The case of methylmercury in fish and seafood. Crit. Rev. Toxicol..

[27] Mazahery H., Stonehouse W., Delshad M., Kruger M.C., Conlon C.A., Beck K.L., von Hurst P.R. (2017). Relationship between Long Chain n-3 Polyunsaturated Fatty Acids and Autism Spectrum Disorder: Systematic Review and Meta-Analysis of Case-Control and Randomised Controlled Trials. Nutrients.

[28] Madore C., Leyrolle Q., Lacabanne C., Benmamar-Badel A., Joffre C., Nadjar A., Layé S. (2016). Neuroinflammation in Autism: Plausible Role of Maternal Inflammation, Dietary Omega 3, and Microbiota. Neural Plast..

[29] Scott J.A. (2020). The first 1000 days: A critical period of nutritional opportunity and vulnerability. Nutr. Diet..

[30] Holmes P., James K.A., Levy L.S. (2009). Is low-level environmental mercury exposure of concern to human health?. Sci. Total Environ..

[31] Clarkson T.W., Jayesh V.B., Ballatori N. (2007). Mechanisms of mercury disposition in the body. Am. J. Ind. Med..

[32] Persson M., Fagt S., Nauta M.J. (2018). Personalised fish intake recommendations: The effect of background exposure on optimisation. Br. J. Nutr..

[33] Quality Assessment Tool for Observational Cohort and Cross-Sectional Studies. https://www.nhlbi.nih.gov/health-topics/study-quality-assessment-tools.

[34] Lederer D.J., Bell S.C., Branson R.D., Chalmers J.D., Marshall R., Maslove D.M., Ost D.E., Punjabi N.M., Schatz M., Smyth A.R. (2018). Control of Confounding and Reporting of Results in Causal Inference Studies. Guidance for Authors from Editors of Respiratory, Sleep, and Critical Care Journals. Ann. Am. Thorac. Soc..

[35] Chin-Lun Hung G., Hahn J., Alamiri B., Buka S.L., Goldstein J.M., Laird N., Nelson C.A., Smoller J.W., Gilman S.E. (2015). Socioeconomic disadvantage and neural development from infancy through early childhood. Int. J. Epidemiol..

[36] Lim S., Ha M., Hwang S.-S., Son M., Kwon H.-J. (2015). Disparities in Children’s Blood Lead and Mercury Levels According to Community and Individual Socioeconomic Positions. Int. J. Environ. Res. Public Health.

[37] Wehby G.L., Prater K., McCarthy A.M., Castilla E.E., Murray J.C. (2011). The Impact of Maternal Smoking during Pregnancy on Early Child Neurodevelopment. J. Hum. Cap..

[38] Gaxiola-Robles R., Bentzen R., Zenteno-Savín T., Labrada-Martagón V., Castellini J.M., Celis A., O’Hara T., Méndez-Rodríguez L.C. (2014). Marine diet and tobacco exposure affects mercury concentrations in pregnant women (I) from Baja California Sur, Mexico. Toxicol. Rep..

[39] Avella-Garcia C.B., Julvez J. (2014). Seafood Intake and Neurodevelopment: A Systematic Review. Curr. Environ. Health Rep..

[40] Moon S.-W., Gwak J.-I., Park Y.-H. (2016). The Effect of Smoking and Second-Hand Smoking on the Concentration of Mercury, Lead and Cadmium in the Blood: Based on the Fifth Korea National Health and Nutrition Examination Survey. Korean J. Fam. Pract..

[41] Næss S., Kjellevold M., Dahl L., Nerhus I., Midtbø L.K., Bank M.S., Rasinger J.D., Markhus M.W. (2020). Effects of seafood consumption on mercury exposure in Norwegian pregnant women: A randomized controlled trial. Environ. Int..

[42] Sharp M., DeMauro S.B. (2017). Counterbalanced Comparison of the BSID-II and Bayley-III at Eighteen to Twenty-two Months Corrected Age. J. Dev. Behav. Pediatr..

[43] Suzuki K., Nakai K., Sugawara T., Nakamura T., Ohba T., Shimada M., Hosokawa T., Okamura K., Sakai T., Kurokawa N. (2010). Neurobehavioral effects of prenatal exposure to methylmercury and PCBs, and seafood intake: Neonatal behavioral assessment scale results of Tohoku study of child development. Environ. Res..

[44] Rothenberg S.E., Yu X., Liu J., Biasini F.J., Hong C., Jiang X., Nong Y., Cheng Y., Korrick S.A. (2016). Maternal methylmercury exposure through rice ingestion and offspring neurodevelopment: A prospective cohort study. Int. J. Hyg. Environ. Health.

[45] Marques R.C., Dórea J.G., Bernardi J.V., Bastos W.R., Malm O. (2009). Prenatal and postnatal mercury exposure, breastfeeding and neurodevelopment during the first 5 years. Cogn. Behav. Neurol..

[46] Nyanza E.C., Bernier F.P., Martin J.W., Manyama M., Hatfield J., Dewey D. (2021). Effects of prenatal exposure and co-exposure to metallic or metalloid elements on early infant neurodevelopmental outcomes in areas with small-scale gold mining activities in Northern Tanzania. Environ. Int..

[47] Xu Y., Khoury J.C., Sucharew H., Dietrich K., Yolton K. (2016). Low-level gestational exposure to mercury and maternal fish consumption: Associations with neurobehavior in early infancy. Neurotoxicol. Teratol..

[48] Kim Y., Ha E.-H., Park H., Ha M., Kim Y., Hong Y.-C., Lee E.J., Kim H., Chang N., Kim B.-N. (2018). Prenatal mercury exposure, fish intake and neurocognitive development during first three years of life: Prospective cohort mothers and Children’s environmental health (MOCEH) study. Sci. Total Environ..

[49] Polanska K., Hanke W., Sobala W., Trzcinka-Ochocka M., Ligocka D., Brzeznicki S., Strugala-Stawik H., Magnus P. (2013). Developmental Effects of Exposures to Environmental Factors: The Polish Mother and Child Cohort Study. Biomed. Res. Int..

[50] Davidson P.W., Strain J.J., Myers G.J., Thurston S.W., Bonham M.P., Shamlaye C.F., Stokes-Riner A., Wallace J.M., Robson P.J., Duffy E.M. (2008). Neurodevelopmental effects of maternal nutritional status and exposure to methylmercury from eating fish during pregnancy. Neurotoxicology.

[51] Lin C.C., Chen Y.C., Su F.C., Lin C.M., Liao H.F., Hwang Y.H., Hsieh W.S., Jeng S.F., Su Y.N., Chen P.C. (2013). In utero exposure to environmental lead and manganese and neurodevelopment at 2 years of age. Environ. Res.

[52] Johnson S., Marlow N. (2006). Developmental screen or developmental testing?. Early Hum. Dev..

[53] Shah-Kulkarni S., Lee S., Jeong K.S., Hong Y.-C., Park H., Ha M., Kim Y., Ha E.-H. (2020). Prenatal exposure to mixtures of heavy metals and neurodevelopment in infants at 6 months. Environ. Res..

[54] Strain J.J., Yeates A.J., van Wijngaarden E., Thurston S.W., Mulhern M.S., McSorley E.M., Watson G.E., Love T.M., Smith T.H., Yost K. (2015). Prenatal exposure to methyl mercury from fish consumption and polyunsaturated fatty acids: Associations with child development at 20 mo of age in an observational study in the Republic of Seychelles. Am. J. Clin. Nutr..

[55] Boucher O., Muckle G., Jacobson J.L., Carter R.C., Kaplan-Estrin M., Ayotte P., Dewailly É., Jacobson S.W. (2014). Domain-specific effects of prenatal exposure to PCBs, mercury, and lead on infant cognition: Results from the Environmental Contaminants and Child Development Study in Nunavik. Environ. Health Perspect..

[56] Jedrychowski W., Perera F., Jankowski J., Rauh V., Flak E., Caldwell K.L., Jones R.L., Pac A., Lisowska-Miszczyk I. (2007). Fish consumption in pregnancy, cord blood mercury level and cognitive and psychomotor development of infants followed over the first three years of life: Krakow epidemiologic study. Environ. Int..

[57] Kim S., Eom S., Kim H.J., Lee J.J., Choi G., Choi S., Kim S., Kim S.Y., Cho G., Kim Y.D. (2018). Association between maternal exposure to major phthalates, heavy metals, and persistent organic pollutants, and the neurodevelopmental performances of their children at 1 to 2years of age- CHECK cohort study. Sci. Total Environ..

[58] Lederman S.A., Jones R.L., Caldwell K.L., Rauh V., Sheets S.E., Tang D., Viswanathan S., Becker M., Stein J.L., Wang R.Y. (2008). Relation between cord blood mercury levels and early child development in a World Trade Center cohort. Environ. Health Perspect..

[59] Llop S., Guxens M., Murcia M., Lertxundi A., Ramon R., Riano I., Rebagliato M., Ibarluzea J., Tardon A., Sunyer J. (2012). Prenatal exposure to mercury and infant neurodevelopment in a multicenter cohort in Spain: Study of potential modifiers. Am. J. Epidemiol..

[60] Yi Y.G., Sung I.Y., Yuk J.S. (2018). Comparison of Second and Third Editions of the Bayley Scales in Children with Suspected Developmental Delay. Ann. Rehabil. Med..

[61] Barbone F., Rosolen V., Mariuz M., Parpinel M., Casetta A., Sammartano F., Ronfani L., Vecchi Brumatti L., Bin M., Castriotta L. (2019). Prenatal mercury exposure and child neurodevelopment outcomes at 18 months: Results from the Mediterranean PHIME cohort. Int. J. Hyg. Environ. Health.

[62] Castriotta L., Rosolen V., Biggeri A., Ronfani L., Catelan D., Mariuz M., Bin M., Brumatti L.V., Horvat M., Barbone F. (2020). The role of mercury, selenium and the Se-Hg antagonism on cognitive neurodevelopment: A 40-month follow-up of the Italian mother-child PHIME cohort. Int. J. Hyg. Environ. Health.

[63] Nišević J.R., Prpić I., Kolić I., Baždarić K., Tratnik J.S., Prpić I., Mazej D., Špirić Z., Barbone F., Horvat M. (2019). Combined prenatal exposure to mercury and LCPUFA on newborn’s brain measures and neurodevelopment at the age of 18 months. Environ. Res..

[64] Snoj Tratnik J., Falnoga I., Trdin A., Mazej D., Fajon V., Miklavčič A., Kobal A.B., Osredkar J., Sešek Briški A., Krsnik M. (2017). Prenatal mercury exposure, neurodevelopment and apolipoprotein E genetic polymorphism. Environ. Res..

[65] Valent F., Mariuz M., Bin M., Little D., Mazej D., Tognin V., Tratnik J., McAfee A.J., Mulhern M.S., Parpinel M. (2013). Associations of prenatal mercury exposure from maternal fish consumption and polyunsaturated fatty acids with child neurodevelopment: A prospective cohort study in Italy. J. Epidemiol..

[66] Wang J., Wu W., Li H., Cao L., Wu M., Liu J., Gao Z., Zhou C., Liu J., Yan C. (2019). Relation of prenatal low-level mercury exposure with early child neurobehavioral development and exploration of the effects of sex and DHA on it. Environ. Int..

[67] Tatsuta N., Nakai K., Murata K., Suzuki K., Iwai-Shimada M., Kurokawa N., Hosokawa T., Satoh H. (2014). Impacts of prenatal exposures to polychlorinated biphenyls, methylmercury, and lead on intellectual ability of 42-month-old children in Japan. Environ. Res..

[68] Freire C., Amaya E., Gil F., Fernández M.F., Murcia M., Llop S., Andiarena A., Aurrekoetxea J., Bustamante M., Guxens M. (2018). Prenatal co-exposure to neurotoxic metals and neurodevelopment in preschool children: The Environment and Childhood (INMA) Project. Sci. Total Environ..

[69] Oken E., Radesky J.S., Wright R.O., Bellinger D.C., Amarasiriwardena C.J., Kleinman K.P., Hu H., Gillman M.W. (2008). Maternal Fish Intake during Pregnancy, Blood Mercury Levels, and Child Cognition at Age 3 Years in a US Cohort. Am. J. Epidemiol..

[70] Oken E., Wright R.O., Kleinman K.P., Bellinger D., Amarasiriwardena C.J., Hu H., Rich-Edwards J.W., Gillman M.W. (2005). Maternal fish consumption, hair mercury, and infant cognition in a U.S. Cohort. Environ. Health Perspect..

[71] Tatsuta N., Murata K., Iwai-Shimada M., Yaginuma-Sakurai K., Satoh H., Nakai K. (2017). Psychomotor Ability in Children Prenatally Exposed to Methylmercury: The 18-Month Follow-Up of Tohoku Study of Child Development. Tohoku J. Exp. Med..

[72] Prpic I., Milardovic A., Vlasic-Cicvaric I., Spiric Z., Radic Nisevic J., Vukelic P., Snoj Tratnik J., Mazej D., Horvat M. (2017). Prenatal exposure to low-level methylmercury alters the child’s fine motor skills at the age of 18 months. Environ. Res..

[73] Hu Y., Chen L., Wang C., Zhou Y., Zhang Y., Wang Y., Shi R., Gao Y., Tian Y. (2016). Prenatal low-level mercury exposure and infant neurodevelopment at 12 months in rural northern China. Environ. Sci. Pollut. Res. Int..

[74] Daniels J.L., Longnecker M.P., Rowland A.S., Golding J. (2004). Fish intake during pregnancy and early cognitive development of offspring. Epidemiology.

[75] Wu J., Ying T., Shen Z., Wang H. (2014). Effect of low-level prenatal mercury exposure on neonate neurobehavioral development in China. Pediatr. Neurol..

[76] Golding J., Gregory S., Iles-Caven Y., Hibbeln J., Emond A., Taylor C.M. (2016). Associations between prenatal mercury exposure and early child development in the ALSPAC study. Neurotoxicology.

[77] Valko M., Morris H., Cronin M.T. (2005). Metals, toxicity and oxidative stress. Curr. Med. Chem..

[78] Chapman L., Chan H.M. (2000). The influence of nutrition on methyl mercury intoxication. Environ. Health Perspect..

[79] Philibert A., Fillion M., Mergler D. (2020). Mercury exposure and premature mortality in the Grassy Narrows First Nation community: A retrospective longitudinal study. Lancet Planet. Health.

[80] Grandjean P., Weihe P., White R.F., Debes F., Araki S., Yokoyama K., Murata K., Sørensen N., Dahl R., Jørgensen P.J. (1997). Cognitive Deficit in 7-Year-Old Children with Prenatal Exposure to Methylmercury. Neurotoxicol. Teratol..

[81] Taylor C.M., Golding J., Emond A.M. (2014). Lead, cadmium and mercury levels in pregnancy: The need for international consensus on levels of concern. J. Dev. Orig. Health Dis..

[82] Rice D.C., Schoeny R., Mahaffey K. (2003). Methods and rationale for derivation of a reference dose for methylmercury by the US EPA. Risk Anal. Int. J..

[83] Rice D.C. (2004). The US EPA reference dose for methylmercury: Sources of uncertainty. Environ. Res..

[84] Mahaffey K.R., Clickner R.P., Bodurow C.C. (2004). Blood organic mercury and dietary mercury intake: National Health and Nutrition Examination Survey, 1999 and 2000. Environ. Health Perspect..

[85] Alhibshi E. (2012). Subclinical neurotoxicity of mercury: A behavioural, molecular mechanisms and therapeutic perspective. Res. J. Pharm. Biol. Chem. Sci..

[86] Taylor C.M., Emmett P.M., Emond A.M., Golding J. (2018). A review of guidance on fish consumption in pregnancy: Is it fit for purpose?. Public Health Nutr..

[87] Saavedra S., Fernández-Recamales Á., Sayago A., Cervera-Barajas A., González-Domínguez R., Gonzalez-Sanz J.D. (2022). Impact of dietary mercury intake during pregnancy on the health of neonates and children: A systematic review. Nutr. Rev..

[88] Hibbeln J.R., Spiller P., Brenna J.T., Golding J., Holub B.J., Harris W.S., Kris-Etherton P., Lands B., Connor S.L., Myers G. (2019). Relationships between seafood consumption during pregnancy and childhood and neurocognitive development: Two systematic reviews. Prostaglandins Leukot. Essent. Fat. Acids.

[89] Grandjean P., Budtz-Jørgensen E., Jørgensen P.J., Weihe P. (2005). Umbilical cord mercury concentration as biomarker of prenatal exposure to methylmercury. Environ. Health Perspect..

[90] Kozikowska I., Binkowski Ł.J., Szczepańska K., Sławska H., Miszczuk K., Śliwińska M., Łaciak T., Stawarz R. (2013). Mercury concentrations in human placenta, umbilical cord, cord blood and amniotic fluid and their relations with body parameters of newborns. Environ. Pollut..

