# Supplementary material for: Prenatal Mercury Exposure and Neurodevelopment up to the Age of 5 Years: A Systematic Review
Source: Int J Environ Res Public Health. 2022 Feb 10;19(4):1976. doi: 10.3390/ijerph19041976 (PMC8871549; doi:10.3390/ijerph19041976)
Supplement: Supplementary file 1 [file ijerph-19-01976-s001.zip › ijerph-1464367-supplementary.pdf]

Appendices

Part 1: Changes to the protocol.

Part 2: Search terms.

Part 3: Fields used in data extraction.

Part 4. Quality assessment tool.

Part 5. Measures of neurodevelopmental functioning in the included studies – further details.

Part 6. Mercury exposure characteristics in included studies.

Part 7. Results of NIH Quality Assessment.

Part 8. Results from all studies.

Part 1: Changes to the protocol.

Change	Rationale
Divided review into two projects to be published separately: 1. Mercury and fetal growth. 2. Mercury and neurodevelopment.	More studies were found than expected, and if all outcomes were discussed in a single paper it would not be possible to adequately discuss all the papers found.
Additional criteria:<br>Include: Studies that used multivariable statistical methods.<br>Exclude: Studies that used only univariable methods such as correlation, *t*-test, etc.	A wide range of potential factors can confound the relationship between mercury and fetal growth. The results of univariable analyses could not answer the research question of this review in a meaningful way.
Additional criteria:<br>Exclude: Studies of Ethylmercury (etHg).	Exposure to ethylmercury is primarily through vaccines, and this has been thoroughly studied elsewhere.
Replaced the Newcastle Ottawa Scale with NIH QA Tool for Observational Cohort and Cross-Sectional Studies.	The NOS scale was not suitable for many of the included studies. The NIH QA Tool proved to be more sensitive and could be applied to both cohort and cross-sectional studies.
Did not meta-analyse study results.	This was not possible because of the wide range of exposures and types of model estimate reported.

Part 2: Search terms.

The following search strategy was adapted to each database.

Population		Exposure		Outcome		Filter
Fetal	**AND**	Mercury	**AND**	neurodevelopment*	**NOT**	Animals
Prenatal		Methylmercury		cognition		
Pre-natal				cognitive		
Gestation				intelligence		
Post-natal				memory		
Postnatal				attention		
Neonatal				communication		
Infant				language		
Infancy				speech		
Baby				neurobehavi*r		
Pre-school				motor		
Preschool				coordination		
Early child*				co-ordination		
Early years				social develop*		
Pregnancy				emotional develop*		
Maternal				developmental delay		

<u>Pubmed</u>

("fetal"[Title/Abstract] OR "prenatal"[Title/Abstract] OR "pre-natal"[Title/Abstract] OR "gestation"[Title/Abstract] OR "post-natal"[Title/Abstract] OR "postnatal"[Title/Abstract] OR "neonatal"[Title/Abstract] OR "infant"[Title/Abstract] OR "infancy"[Title/Abstract] OR "baby"[Title/Abstract] OR "pre-school" [Title/Abstract] OR "preschool"[Title/Abstract] OR "early child*"[Title/Abstract] OR "early years"[Title/Abstract] OR "pregnancy"[Title/Abstract] OR "maternal"[Title/Abstract]) AND ("methylmercury compounds"[MeSH Terms] OR "mercury"[MeSH Terms]) AND ("neurodevelopment*"[Title/Abstract] OR "cognition"[MeSH Terms] OR "cognitive"[Title/Abstract] OR "intelligence"[MeSH Terms] OR "memory"[MeSH Terms] OR "attention"[MeSH Terms] OR "communication" [MeSH Terms] OR "language development"[MeSH Terms] OR "speech"[MeSH Terms] OR "motor skills"[MeSH Terms] OR "psychomotor Performance"[MeSH Terms] OR "neurobehavi*r"[Title/Abstract] OR "social develop*"[Title/Abstract] OR "emotional development" [Title/Abstract] OR "developmental delay"[Title/Abstract])

+ filter: humans.

<u>Scopus</u>

TITLE-ABS (fetal OR prenatal OR "pre-natal" OR gestation OR "post-natal" OR postnatal OR neonatal OR infant OR infancy OR baby OR "pre-school" OR

preschool OR "early child*" OR "early years" OR maternal OR pregnancy) AND TITLE-ABS ("neurodevelopment*" OR cognition OR cognitive OR intelligence OR memory OR attention OR communication OR language OR speech OR neurobehavi*r OR motor OR coordination OR "co-ordination" OR "social develop*" OR "emotional develop*" OR "developmental delay") AND TITLE-ABS (mercury OR methylmercury) AND (LIMIT-TO (EXACTKEYWORD , "Human")) AND (EXCLUDE (EXACTKEYWORD , "Animals"))

<u>Embase</u>

((fetal OR prenatal OR "pre-natal" OR gestation OR "post-natal" OR postnatal OR neonatal OR infant OR infancy OR baby OR "pre-school" OR preschool OR "early child*" OR "early years" OR maternal OR pregnancy) and (exp mercury/ OR exp methylmercury/) and ("neurodevelopment*" OR cognition OR cognitive OR intelligence OR memory OR attention OR communication OR language OR speech OR neurobehavi*r OR motor OR coordination OR "co-ordination" OR "social develop*" OR "emotional develop*" OR "developmental delay")).ti,ab. not ((exp animal/ or nonhuman/) not exp human/)

<u>PsycINFO</u>

((fetal OR prenatal OR "pre-natal" OR gestation OR "post-natal" OR postnatal OR neonatal OR infant OR infancy OR baby OR "pre-school" OR preschool OR "early child*" OR "early years" OR maternal OR pregnancy) and ((exp "mercury (metal)"/) OR (methylmercury.ti,ab.) and ("neurodevelopment*" OR cognition OR cognitive OR intelligence OR memory OR attention OR communication OR language OR speech OR neurobehavi*r OR motor OR coordination OR "co-ordination" OR "social develop*" OR "emotional develop*" OR "developmental delay")).ti,ab.

Part 3: Fields used in data extraction.

Author

Year

Study design

Country of origin

Recruitment criteria

Total study sample size

Exposure source

Timing of exposure

Mean / SD of exposure

Median / IQR of exposure

Exposure units

Exposure analysis method

Exposure analysis LoD

Outcome

Timing of outcome

Model sample size

Model estimate type

Model coefficient

Model coefficient confidence intervals

Model coefficient p-value

Adjustment set

Part 4. Quality assessment tool

**NIH Quality assessment tool for observational cohort and cross-sectional studies**<br>**Website:** https://www.nhlbi.nih.gov/health-topics/study-quality-assessment-tools			
Major Components	Yes	No	Other (NA, NR, CD)
1. Was the research question or objective in this paper clearly stated?			
2. Was the study population clearly specified and defined?			
3. Was the participation rate of eligible persons at least 50%?			
4. Were all the subjects selected or recruited from the same or similar populations (including the same time period)? Were inclusion and exclusion criteria for being in the study prespecified and applied uniformly to all participants?			
5. Was a sample size justification, power description, or variance and effect estimates provided?			
6. For the analyses in this paper, were the exposure(s) of interest measured prior to the outcome(s) being measured?			
7. Was the timeframe sufficient so that one could reasonably expect to see an association between exposure and outcome if it existed?			
8. For exposures that can vary in amount or level, did the study examine different levels of the exposure as related to the outcome (e.g., categories of exposure, or exposure measured as continuous variable)?			
9. Were the exposure measures (independent variables) clearly defined, valid, reliable, and implemented consistently across all study participants?			
10. Was the exposure(s) assessed more than once over time?**(1)**			

11. Were the outcome measures (dependent variables) clearly defined, valid, reliable, and implemented consistently across all study participants?			
12. Were the outcome assessors blinded to the exposure status of participants?			
13. Was loss to follow-up after baseline 20% or more?			
14. Were key potential confounding variables measured and adjusted statistically for their impact on the relationship between exposure(s) and outcome(s)? **(2)**			
15. Were multivariable models based on prior theory, with covariates selected based on theory or evidence that they may be confounders or competing exposures? **(3)**			
16. Were the results of all analyses described in the study methods reported, including results which did not meet a threshold of statistical significance? **(3)**			

(1) This question was not relevant to the type of studies included in this review. To make it more useful it was taken to also include whether the study reported quality control methods when analysing mercury concentrations.

(2) Key confounders: Maternal socio-economic status or education, fish or fatty acid intake, maternal smoking status. Based on confounders identified in:

Nagpal N, Bettiol S, Isham A, Hoang H, Crocombe L. 2017. A review of mercury exposure and health of dental personnel. Safety and Health at Work. 8, pp.1-10.
World Health Organization. 2008. Guidance for identifying populations at risk from mercury exposure. Available online: https://www.who.int/foodsafety/publications/chem/mercuryexposure.pdf

(3) These are additional criteria added to evaluate statistical issues common to perinatal research.

Part 5. Measures of neurodevelopmental functioning in the included studies – further details.

Abbreviation	Name	Summary	Reference for further information
A-not-B	A-not-B test	Delayed memory task. Use of working memory as a reflection of executive function.	Diamond 1990
BSID-II	Bayley Scales of Infant and Toddler Development - Second Edition	Scales of cognitive & language (MDI) and motor (PDI) neurodevelopment.	Yi et al 2018
Bayley-III / BSID-III	Bayley Scales of Infant and Toddler Development–Third Edition	Scales of cognitive, language, and motor neurodevelopment, with standardised subscales.	Yi et al 2018
CDI	MacArthur-Bates Communicative Development Inventories	Measure of social communication and early language development.	Hellman et al 2005
CDIIT	Comprehensive Developmental Inventory for Infants and Toddlers	Measure of the global, cognitive, language, motor, gross motor, fine motor, social, self help, and behavioural development of children 3 to 71 months old.	Wang et al 1998
DDST	Denver Developmental Screening Test	Assessment of child personal-social, fine motor, language, and gross motor development.	Sciarillo et al 1986
DDST (modified)	Modified version of the Denver Developmental Screening Test	Assessment of child social skills, fine motor, gross motor, and language and communication indicators. Modified from DDST.	Golding et al 2016
FTII	Fagan Test of Infant Intelligence	Assessment of visual novelty preference and fixation duration, designed to reflect memory and speed of processing.	Boucher et al 2014
GDS	Gesell developmental schedules	Measure of child's motor, language, personal-social, and adaptive behaviour.	Ball 1977
K-ABC	Kaufman Assessment Battery for Children	Measures the cognitive processing of children from age 3 and above.	Kaufman et al 1987
K-BSID-II	Korean adapted version of Bayley Scales of Infant Development II	Korean language version of the BSID-II.	Shah-Kulkarni et al 2020
KSPD	Kyoto Scale of Psychological Development	Test measuring fine and gross motor skills, cognitive-adaptive development, and language and social development.	Koyama et al 2009
MCDI	MacArthur Communicative Development Inventory	Parent completed assessment of children's language and communication skills.	Feldman et al 2000
MDAT	Malawi Developmental Assessment Tool	Culturally-specific tool to assess gross motor, fine motor, language, and social domains of development for children up to 6 years old.	Nyanza et al 2021
MSCA	McCarthy Scales of Children's Abilities	Instrument used to measure cognitive development across verbal, quantitative, perceptual-performance, memory, motor, and general cognitive scales.	Nagle 1979
NBAS	Neonatal Behavioral Assessment Scale	Assessment of infants from birth to 4 months old, measures autonomic control, motor function, social interaction, and consciousness.	Costa et al 2010
NBNA	Neonatal Behavioral Neurological Assessment	Based of NBAS. Measure of the behavioural neurological activity of newborns, with assessment of behavioural response, passive tone reaction, active tone reaction, reflexes, and general activity.	Bao et al 1993
NNNS	NICU Network Neurobehavioral Scale	Assessment of the neurobehavioural status of infants, designed to identify potential neurodevelopmental delay.	Provenzi et al 2018

PPVT	Peabody Picture Vocabulary Test	Test designed to assess a child's receptive (auditory) vocabulary through a visual memory task.	Hoffman et al 2012
SMS	Social Maturity Scale (Vineland)	Tool used to measure social and adaptive behaviour.	Raggio et al 1993
VRM	Visual recognition memory	Test of infant recognition and novelty preference, intended to measure cognitive development.	Oken et al 2005
WPPSI-R	Wechsler Preschool and Primary Scales of Intelligence-Revised	Scale of IQ (performance, verbal, full) designed for children aged 3 years to 7 years and 3 months.	Faust et al 1991
WRAVMA	Wide Range Assessment of Visual Motor Abilities	Measures visual motor skills of children aged 3 and above, using drawing, matching, and pegboard tasks.	Obler et al 2011

Part 6. Mercury exposure characteristics in included studies.

			Mercury concentrations					
**Study (year)**	**Country**	**Exposure**	**Mean (SD)**	**Median (IQR)**	**Units**	**Mercury analysis method**	**Limits of detection**	**Timing of exposure**
Barbone et al (2019)	Italy, Slovenia, Croatia, Greece	Hair<br>Whole blood<br>Umbilical cord	997 (1035)<br>3.2 (3.4)<br>5.2 (5.0)	704<br>2.4<br>3.6	ng/g	CVAAS	Blood & cord: < 0.1 ng/mL<br>Hair: < 1 ng/g	20 weeks - delivery<br>20 weeks - delivery<br>At delivery
Boucher et al (2014)	Canada	Umbilical cord	22.5 (16.6)	17	µg/L	CVAAS	1.0 nmol/L	At delivery
Castriotta et al (2020)	Italy	Umbilical cord	3.4 (3.8)	2.4	ng/g	CVAAS		20-32 weeks gestation
Daniels et al (2004)	United Kingdom	Umbilical cord	0.01* (0.4)		µg/g	CVAAS		At delivery
Davidson et al (2008)	Republic of Seychelles	Hair	5.7 (3.7)		µg/g	CVAAS		At delivery
Freire et al (2018)	Spain	Placenta	57.6	0.025 (12.9)	ng/g	CVAAS	0.0156 ng/g	At delivery
Golding et al (2016)	United Kingdom	Whole blood		1.86	µg/L	ICP-DRC-MS	0.24 µg/L	11 weeks gestation
Hu et al (2016)	China	Whole blood<br>Umbilical cord	0.81 (0.41)<br>1.37 (0.73)		µg/L	CVAAS	0.3 µg/L	At delivery
Jedrychowski et al (2007)	Poland	Umbilical cord		0.9	µg/L	CVAAS		At delivery
Kim S et al (2008)	South Korea	Whole blood<br>Umbilical cord		4.5 (3.5)<br>7.7 (6.2)	µg/L			At delivery
Kim Y et al (2018)	South Korea	Whole blood<br>Whole blood<br>Umbilical cord	3.71*<br>3.55*<br>5.1*		µg/L	CVAAS	0.07 µg/L	12-20 weeks gestation<br>28-42 weeks gestation<br>At delivery
Lederman et al (2008)	USA	Umbilical cord	7.82 (9.71)	4.3	µg/L	ICP-MS		At delivery
Lin et al (2013)	Taiwan	Umbilical cord	14.9 (9.42)	12.2	µg/L	ICP-MS	0.28 µg/L	At delivery

Llop et al (2012)	Spain	Umbilical cord	8.4*		µg/L	AAS		At delivery
Marques et al (2009)	Brazil	Hair				CVAAS		At delivery<br>6 months
Nisevic et al (2019)	Croatia, Italy	Umbilical cord			µg/L			At delivery
Nyanza et al (2020)	Tanzania	Whole blood		1.2 (0.9)	µg/L	ICP-MS	0.012 µg/L	16-27 weeks gestation
Oken et al (2005)	USA	Hair	0.55		µg/g	CVAAS		At delivery
Oken et al (2008)	USA	Erythrocyte	3.8 (3.8)		ng/g	CVAAS		2nd trimester
Polanska et al (2013)	Poland	Hair	0.3 (0.2)	0.2	µg/g	CVAAS		30-34 weeks gestation
Prpic et al (2017)	Croatia	Umbilical cord		2.98 (4.2)	ng/g	CVAAS	0.02 ng/g	At delivery
Rothenberg et al (2016)	China	Whole blood<br>Hair	1.2*<br>0.47*		µg/g	AAS	0.0095 µg/g	At delivery
Shah-Kulkarni et al (2020)	South Korea	Whole blood<br>Whole blood<br>Umbilical cord	3.30*<br>3.13*<br>5.21*	2.23 (2)<br>3.13 (2.01)<br>5.33 (3.16)	µg/L	CVAAS	0.07 µg/L	12-20 weeks gestation<br>>28 weeks gestation<br>At delivery
SnojTratnik et al (2017)	Slovenia, Croatia	Hair<br>Umbilical cord	361*<br>2.05*		ng/g	CVAAS	1.0 ng/g<br>0.1 ng/mL	34 weeks gestation / at delivery<br>At delivery
Strain et al (2015)	Republic of Seychelles	Hair	3.92* (3.46)		µg/g	AAS		At delivery
Suzuki et al (2010)	Japan	Hair	2.22 (1.16)	1.96	µg/g	CVAAS	0.1 ng/g	At delivery
Tatsuta et al (2014)	Japan	Umbilical cord		10.1	ng/g	CVAAS		At delivery
Tatsuta et al (2017)	Japan	Umbilical cord		15.7	ng/g	CVAAS		At delivery
Valent et al (2013)	Italy	Hair<br>Umbilical cord	1061 (1028)<br>5.5 (4.8)	788 (600)<br>2.5 (2.8)	ng/g	CVAAS		20-22 weeks gestation<br>At delivery
Wang et al (2019)	China	Umbilical cord	2.0*		µg/L	CVAAS	0.01 µg/l	At delivery

Wu et al (2014)	China	Umbilical cord	7.92 (4.69)	7.62 (6.44)	µg/L	CVAAS		At delivery
Xu et al (2016)	USA	Whole blood<br>Umbilical cord	0.64<br>0.72		µg/L	ICP-MS	0.2 µg/L	16 weeks - delivery<br>At delivery
* Geometric mean								

Part 7. Results of NIH Quality Assessment.

NA: Not applicable.

NR: Not reported.

Study (year)	1	2	3	4	5	6	7	8	9	10	11	12	13	14	15	16	Total
Barbone et al (2019)	1	1	NR	NR	1	1	1	1	1	1	1	1	0	1	0	1	12
Boucher et al (2014)	1	1	NR	1	1	1	1	1	1	0	1	1	1	1	1	1	14
Castriotta et al (2020)	1	1	NA	1	1	1	1	1	1	1	1	1	1	0	0	1	13
Daniels et al (2004)	1	1	1	1	1	1	1	1	1	0	1	1	1	1	1	1	15
Davidson et al (2008)	1	1	1	1	1	1	1	1	1	1	1	1	1	0	1	1	15
Freire et al (2018)	1	1	NR	1	1	1	1	0	1	1	1	1	0	0	0	1	11
Golding et al (2016)	1	1	1	1	1	1	1	1	1	1	1	1	NR	1	1	1	15
Hu et al (2016)	1	1	NA	1	1	1	1	1	1	1	1	1	1	1	1	1	15
Jedrychowski et al (2007)	1	1	NR	1	1	1	1	0	1	0	1	1	NR	0	1	1	11
Kim S et al (2008)	1	1	NR	1	1	1	1	1	1	1	1	1	1	0	1	0	13
Kim Y et al (2018)	1	1	NR	1	1	1	1	1	1	1	1	1	1	1	1	1	15
Lederman et al (2008)	1	1	1	1	1	1	1	1	1	1	1	1	1	0	1	1	15
Lin et al (2013)	1	1	NR	1	0	1	1	0	1	0	1	1	0	1	1	1	11
Llop et al (2012)	1	1	NR	1	1	1	1	1	1	0	1	1	1	1	0	1	13
Marques et al (2009)	1	0	NR	NR	0	1	1	1	1	1	1	1	NR	0	0	1	9
Nisevic et al (2019)	1	1	NR	1	0	1	1	1	1	0	1	1	NR	1	1	1	12
Nyanza et al (2020)	1	1	NR	1	1	1	1	1	1	1	1	1	0	0	1	1	13
Oken et al (2005)	1	1	1	1	1	1	1	1	1	1	1	1	0	0	1	1	14
Oken et al (2008)	1	1	1	1	1	1	1	1	1	1	1	1	1	1	1	1	16
Polanska et al (2013)	1	1	NR	1	1	1	1	1	1	0	1	1	0	0	0	0	10
Prpic et al (2017)	1	1	NR	1	0	1	1	1	1	1	1	1	NR	1	1	0	12
Rothenberg et al (2016)	1	1	NR	1	1	1	1	1	1	1	1	1	0	1	0	1	13
Shah-Kulkarni et al (2020)	1	1	NR	1	1	1	1	1	1	1	1	1	NR	0	1	1	13
SnojTratnik et al (2017)	1	1	NR	1	0	1	1	1	1	0	1	1	0	0	0	1	10
Strain et al (2015)	1	1	NR	1	1	1	1	1	1	0	1	1	1	0	1	1	13
Suzuki et al (2010)	1	1	0	1	1	1	1	1	1	1	1	1	1	1	1	0	14

Tatsuta et al (2014)	1	1	1	1	1	1	1	1	1	1	1	1	0	0	1	1	14
Tatsuta et al (2017)	1	1	1	1	1	1	1	1	1	1	1	1	0	1	1	1	15
Valent et al (2013)	1	1	1	1	1	1	1	1	1	0	1	1	1	1	0	1	14
Wang et al (2019)	1	1	NR	1	1	1	1	1	1	1	1	1	0	0	1	1	13
Wu et al (2014)	1	1	1	1	1	1	1	1	1	1	1	1	1	0	1	1	15
Xu et al (2016)	1	1	NR	1	1	1	1	1	1	0	1	1	1	1	0	1	13

Part 8. Results from studies.

Results reported in included studies, arranged by outcome group (blank spaces indicate data was not reported by study).

Cognition / language (BSID:II MDI)													
**Study**	**n**	**Exposure**	**Units**	**Outcome**	**Time of outcome**	**Estimate type**	**Notes**	**Estimate**	**LCI**	**UCI**	**P-value**	**High quality**
Boucher et al (2014)	87	Umbilical cord	ln µg/L	BSID-II: MDI	11 months	RR		0.08	-0.15	0.33		Yes
Davidson et al (2008)	225	Hair	ppm	BSID-II: MDI	30 months	RR		-0.17			0.35	
Davidson et al (2008)	225	Hair	ppm	BSID-II: MDI	9 months	RR		-0.17			0.39	
Jedrychowski et al (2007)	374	Umbilical cord	Hg > 0.90 µg/L	BSID-II: MDI	12 months	RR		-2.78			0.01	
Jedrychowski et al (2007)	353	Umbilical cord	Hg > 0.90 µg/L	BSID-II: MDI	24 months	RR		-1.05			0.42	
Jedrychowski et al (2007)	270	Umbilical cord	Hg > 0.90 µg/L	BSID-II: MDI	36 months	RR		1.09			0.37	
Kim S et al (2008)	88	Umbilical cord	µg/L	BSID-II: MDI	13-24 months	RR		-5.39	-12.46	1.69		
Kim Y et al (2018)	595	Umbilical cord	µg/L	BSID-II: MDI	12 months	RR		-0.04	-0.39	0.32	0.85	Yes
Kim Y et al (2018)	523	Umbilical cord	µg/L	BSID-II: MDI	24 months	RR		-0.03	-0.36	0.30	0.87	Yes
Kim Y et al (2018)	438	Umbilical cord	µg/L	BSID-II: MDI	36 months	RR		0.17	-0.26	0.60	0.43	Yes
Kim Y et al (2018)	662	Umbilical cord	µg/L	BSID-II: MDI	6 months	RR		-0.03	-0.27	0.21	0.80	Yes
Kim Y et al (2018)	763	Whole blood	µg/L	BSID-II: MDI	12 months	RR	early pregnancy	-0.32	-0.89	0.26	0.28	Yes
Kim Y et al (2018)	614	Whole blood	µg/L	BSID-II: MDI	12 months	RR	late pregnancy	-0.07	-0.69	0.55	0.82	Yes
Kim Y et al (2018)	686	Whole blood	µg/L	BSID-II: MDI	24 months	RR	early pregnancy	-0.06	-0.63	0.51	0.83	Yes
Kim Y et al (2018)	564	Whole blood	µg/L	BSID-II: MDI	24 months	RR	late pregnancy	-0.46	-1.034	0.118	0.119	Yes
Kim Y et al (2018)	557	Whole blood	µg/L	BSID-II: MDI	36 months	RR	early pregnancy	-0.28	-0.89	0.32	0.36	Yes

Kim Y et al (2018)	460	Whole blood	µg/L	BSID-II: MDI	36 months	RR	late pregnancy	-0.25	-0.88	0.38	0.43	Yes
Kim Y et al (2018)	847	Whole blood	µg/L	BSID-II: MDI	6 months	RR	early pregnancy	-0.41	-0.81	-0.003	0.05	Yes
Kim Y et al (2018)	689	Whole blood	µg/L	BSID-II: MDI	6 months	RR	late pregnancy	-0.13	-0.56	0.29	0.54	Yes
Lederman et al (2008)	132	Umbilical cord	Ln µg/L	BSID-II: MDI	12 months	RR		-0.53			0.60	
Lederman et al (2008)	131	Umbilical cord	Ln µg/L	BSID-II: MDI	24 months	RR		-2.38			0.07	
Lederman et al (2008)	114	Umbilical cord	Ln µg/L	BSID-II: MDI	36 months	RR		-1.43			0.24	
Llop et al (2012)	1683	Umbilical cord	Ln µg/L	BSID-II: MDI	14 months	RR		0.16	-0.12	0.45		Yes
Rothenberg et al (2016)	270	Hair	Ln µg/g	BSID-II: MDI	12 months	RR		-4.9	-9.7	-0.1		Yes
Shah-Kulkarni et al (2020)	321	Umbilical cord	µg/L	BSID-II: MDI	6 months	RR		0.79	-1.68	3.26		
Shah-Kulkarni et al (2020)	523	Whole blood	µg/L	BSID-II: MDI	6 months	RR	early pregnancy	0.11	-1.79	2.02		
Shah-Kulkarni et al (2020)	467	Whole blood	µg/L	BSID-II: MDI	6 months	RR	late pregnancy	-0.13	-0.61	0.34		
Strain et al (2015)	1241	Hair	µg/g	BSID-II: MDI	20 months	RR		-0.08			0.31	

Cognition

Study	n	Exposure	Units	Outcome	Time of outcome	Estimate type	Notes	Estimate	LCI	UCI	P-value	High quality
Barbone et al (2019)	1083	Hair	ng/g	Bayley-III: Cognitive composite	18 months	RR		0.20	-0.29	0.69		Yes
Barbone et al (2019)	829	Umbilical cord	ng/g	Bayley-III: Cognitive composite	18 months	RR		0.13	-0.39	0.64		Yes
Barbone et al (2019)	636	Whole blood	ng/g	Bayley-III: Cognitive composite	18 months	RR		-0.09	-0.61	0.43		Yes
Boucher et al (2014)	77	Umbilical cord	ln µg/L	A-not-B: 2 correct	11 months	RR		-0.25	-0.46	0.00		Yes
Boucher et al (2014)	77	Umbilical cord	ln µg/L	A-not-B: 3 correct	11 months	RR		-0.22	-0.45	0.03		Yes
Boucher et al (2014)	73	Umbilical cord	ln µg/L	Persevarative errors	11 months	RR		-0.21	-0.53	0.09		Yes

Boucher et al (2014)	89	Umbilical cord	ln µg/L	FTII: Novelty preference	6.5 months	RR		0.00	-0.19	0.19		Yes
Boucher et al (2014)	89	Umbilical cord	ln µg/L	FTIII: Fixation duration	6.5 months	RR		0.13	-0.03	0.29		Yes
Castriotta et al (2020)	323	Umbilical cord	ng/g	Bayley-III: Cognitive composite	40 months	OR: Medium vs Low Hg	90% CI	0.67	0.37	1.20		
Castriotta et al (2020)	323	Umbilical cord	ng/g	Bayley-III: Cognitive composite	40 months	OR: High vs Low HG	90% CI	0.96	0.56	1.66		
Freire et al (2018)	302	Placenta	Binary: Detected Hg	MSCA: Executive function	48-60 months	RR		1.13	-2.66	4.93		
Freire et al (2018)	302	Placenta	Binary: Detected Hg	MSCA: General Cognitive	48-60 months	RR		-0.27	-4.12	3.57		
Freire et al (2018)	302	Placenta	Binary: Detected Hg	MSCA: Memory	48-60 months	RR		-0.42	-4.45	3.61		
Freire et al (2018)	302	Placenta	Binary: Detected Hg	MSCA: Memory span	48-60 months	RR		0.23	-3.7	4.17		
Freire et al (2018)	302	Placenta	Binary: Detected Hg	MSCA: Visual posterior cortex	48-60 months	RR		-1.26	-5.21	2.69		
Lederman et al (2008)	107	Umbilical cord	Ln µg/L	WPPSI-R: Full IQ	48 months	RR		-3.62			0.004	
Lederman et al (2008)	107	Umbilical cord	Ln µg/L	WPPSI-R: Performanc e IQ	48 months	RR		-3.20			0.04	
Lederman et al (2008)	107	Umbilical cord	Ln µg/L	WPPSI-R: Verbal IQ	48 months	RR		-2.87			0.03	
Lin et al (2013)	230	Umbilical cord	Hg ≥ 19.78 µg/L	CDIIT: Cognitive	24 months	RR		0.09			NS	Yes
Nisevic et al (2019)	257	Umbilical cord	µg/L	Bayley-III: Cognitive composite	18 months	RR		0.14			0.34	Yes
Oken et al (2005)	135	Hair	ppm	VRM	6 months	RR		-4.6	-10.3	1.1		

Oken et al (2008)	341	Erythrocyte	ng/g	WRAVMA matching	36 months	RR		-0.2	-0.6	0.2		Yes
Polanska et al (2013)	303	Hair	µg/g	Bayley-III: Cognitive composite	12-24 months	RR		1.6	-4.4	7.6		
Snoj Tratnik et al (2017)	283	Hair	Ln µg/g	Bayley-III: Cognitive composite	16 - 20 months	RR		-1.12	-3.08	0.84	0.26	
Snoj Tratnik et al (2017)	280	Umbilical cord	Ln µg/g	Bayley-III: Cognitive composite	16 - 20 months	RR		-1.41	-3.47	0.66	0.18	
Tatsuta et al (2014)	287	Umbilical cord	Ln ng/g	K-ABC: Mental processing	42 months	RR		1.40	-4.86	7.67		
Tatsuta et al (2014)	287	Umbilical cord	Ln ng/g	K-ABC: Sequential processing	42 months	RR		0.69	-6.66	8.04		
Valent et al (2013)	505	Hair	Ln ng/g	Bayley-III: Cognitive composite	18 months	RR		-0.002			0.997	Yes
Valent et al (2013)	378	Umbilical cord	Ln ng/g	Bayley-III: Cognitive composite	18 months	RR		0.05			0.92	Yes
Wang et al (2019)	236	Umbilical cord	ng/g	Bayley-III: Cognitive composite	21 months	RR		-0.39	-1.18	0.4		
Xu et al (2016)	270	Umbilical cord	µg/L	NNNS: Attention	5 weeks	RR		0.12			0.23	Yes
Xu et al (2016)	344	Whole blood	µg/L	NNNS: Attention	5 weeks	RR		0.15			0.22	Yes

Motor

Study	n	Exposure	Units	Outcome	Time of outcome	Estimate type	Notes	Estimate	LCI	UCI	P-value	High quality
Barbone et al (2019)	1082	Hair	ng/g	Bayley-III: Fine Motor scale	18 months	RR		-0.03	-0.11	0.06		Yes
Barbone et al (2019)	1081	Hair	ng/g	Bayley-III: Gross Motor scale	18 months	RR		-0.01	-0.07	0.05		Yes
Barbone et al (2019)	1083	Hair	ng/g	Bayley-III: Motor	18 months	RR		-0.12	-0.47	0.22		Yes

				developme nt								
Barbone et al (2019)	635	Umbilical cord	ng/g	Bayley-III: Gross Motor scale	18 months	RR		-0.02	-0.08	0.05		Yes
Barbone et al (2019)	892	Umbilical cord	ng/g	Bayley-III: Motor developme nt	18 months	RR		-0.11	-0.47	0.25		Yes
Barbone et al (2019)	636	Whole blood	ng/g	Bayley-III: Fine Motor scale	18 months	RR		0.05	-0.04	0.15		Yes
Barbone et al (2019)	890	Whole blood	ng/g	Bayley-III: Gross Motor scale	18 months	RR		-0.03	-0.09	0.03		Yes
Barbone et al (2019)	636	Whole blood	ng/g	Bayley-III: Motor developme nt	18 months	RR		0.11	-0.25	0.48		Yes
Boucher et al (2014)	87	Umbilical cord	ln µg/L	BSID-II: PDI	11 months	RR		0.01	-0.24	0.25		Yes
Davidson et al (2008)	225	Hair	ppm	BSID-II: PDI	30 months	RR		-0.55			0.04	
Davidson et al (2008)	225	Hair	ppm	BSID-II: PDI	9 months	RR		-0.16			0.34	
Freire et al (2018)	302	Placenta	Binary: Detected Hg	MSCA: Motor	48-60 months	RR		-2.32	-6.29	1.64		
Hu et al (2016)	410	Umbilical cord	Ln µg/L	GDS: Fine motor domain	12 months	RR		-2.62	-7.78	2.55		Yes
Hu et al (2016)	410	Umbilical cord	Ln µg/L	GDS: Gross motor domain	12 months	RR		1.95	-3.08	6.98		Yes
Hu et al (2016)	410	Whole blood	Ln µg/L	GDS: Fine motor domain	12 months	RR		2.69	-3.37	8.74		Yes
Hu et al (2016)	410	Whole blood	Ln µg/L	GDS: Gross motor domain	12 months	RR		3.26	-2.72	9.24		Yes

Jedrychowski et al (2007)	374	Umbilical cord	Hg > 0.90 µg/L	BSID-II: PDI	12 months	RR		-2.33			0.04	
Jedrychowski et al (2007)	350	Umbilical cord	Hg > 0.90 µg/L	BSID-II: PDI	24 months	RR		-1.38			0.2	
Jedrychowski et al (2007)	263	Umbilical cord	Hg > 0.90 µg/L	BSID-II: PDI	36 months	RR		1.15			0.37	
Kim Y et al (2018)	595	Umbilical cord	µg/L	BSID-II: PDI	12 months	RR		0.14	-0.23	0.52	0.45	Yes
Kim Y et al (2018)	523	Umbilical cord	µg/L	BSID-II: PDI	24 months	RR		0.16	-0.17	0.48	0.34	Yes
Kim Y et al (2018)	438	Umbilical cord	µg/L	BSID-II: PDI	36 months	RR		-0.13	-0.55	0.27	0.53	Yes
Kim Y et al (2018)	662	Umbilical cord	µg/L	BSID-II: PDI	6 months	RR		-0.20	-0.45	0.15	0.33	Yes
Kim Y et al (2018)	763	Whole blood	µg/L	BSID-II: PDI	12 months	RR	early pregnancy	0.30	-0.31	0.91	0.34	Yes
Kim Y et al (2018)	614	Whole blood	µg/L	BSID-II: PDI	12 months	RR	late pregnancy	0.27	-0.38	0.93	0.41	Yes
Kim Y et al (2018)	686	Whole blood	µg/L	BSID-II: PDI	24 months	RR	early pregnancy	-0.17	-0.74	0.40	0.556	Yes
Kim Y et al (2018)	564	Whole blood	µg/L	BSID-II: PDI	24 months	RR	late pregnancy	-0.09	-0.67	0.48	0.75	Yes
Kim Y et al (2018)	557	Whole blood	µg/L	BSID-II: PDI	36 months	RR	early pregnancy	-0.11	-0.70	0.47	0.70	Yes
Kim Y et al (2018)	460	Whole blood	µg/L	BSID-II: PDI	36 months	RR	late pregnancy	-0.58	-1.19	0.03	0.06	Yes
Kim Y et al (2018)	847	Whole blood	µg/L	BSID-II: PDI	6 months	RR	early pregnancy	-0.55	-1.05	-0.05	0.03	Yes
Kim Y et al (2018)	689	Whole blood	µg/L	BSID-II: PDI	6 months	RR	late pregnancy	-0.27	-0.78	0.25	0.31	Yes
Lederman et al (2008)	132	Umbilical cord	Ln µg/L	BSID-II: PDI	12 months	RR		-1.39			0.36	
Lederman et al (2008)	130	Umbilical cord	Ln µg/L	BSID-II: PDI	24 months	RR		-2.20			0.10	
Lederman et al (2008)	111	Umbilical cord	Ln µg/L	BSID-II: PDI	36 months	RR		-4.07			0.01	
Lin et al (2013)	230	Umbilical cord	Hg ≥ 19.78 µg/L	CDIT: Fine-motor	24 months	RR		-1.96			NS	
Lin et al (2013)	230	Umbilical cord	Hg ≥ 19.78 µg/L	CDIT: Gross-motor	24 months	RR		2.30			NS	

Lin et al (2013)	230	Umbilical cord	Hg ≥ 19.78 µg/L	CDIT: Motor	24 months	RR		0.03			NS	
Llop et al (2012)	1683	Umbilical cord	Ln µg/L	BSID-II: PDI	14 months	RR		-0.05	-0.79	0.68		Yes
Nisevic et al (2019)	257	Umbilical cord	µg/L	Bayley-III: Fine Motor scale	18 months	RR		-0.07			0.78	Yes
Nisevic et al (2019)	257	Umbilical cord	µg/L	Bayley-III: Gross Motor scale	18 months	RR		0.08			0.71	Yes
Nisevic et al (2019)	257	Umbilical cord	µg/L	Bayley-III: Motor developme nt	18 months	RR		0.01			0.92	Yes
Nyanza et al (2021)	429	Whole blood	µg/L	MDAT: Fine motor	6-12 months	aPR		1.02	0.9	1.1		
Nyanza et al (2021)	429	Whole blood	µg/L	MDAT: Gross motor	6-12 months	aPR		0.9	0.9	1.1		
Oken et al (2008)	341	Erythrocyte	ng/g	WRAVMA drawing	36 months	RR		0.1	-0.2	0.4		Yes
Oken et al (2008)	341	Erythrocyte	ng/g	WRAVMA pegboard	36 months	RR		0.03	-0.3	0.3		Yes
Polanska et al (2013)	303	Hair	µg/g	Bayley-III: Motor developme nt	12-24 months	RR		5.3	-2.2	12.9		
Prpic et al (2017)	135	Umbilical cord	ng/g	Bayley-III: Fine Motor scale	18 months	RR		0.06			0.28	
Rothenberg et al (2016)	270	Hair	Ln µg/g	BSID-II: PDI	12 months	RR		-2.7	-8.3	2.9		Yes
Shah-Kulkarni et al (2020)	321	Umbilical cord	µg/L	BSID-II: PDI	6 months	RR		-1.14	-4.32	2.02		
Shah-Kulkarni et al (2020)	523	Whole blood	µg/L	BSID-II: PDI	6 months	RR	early pregnancy	0.11	-2.31	2.53		
Shah-Kulkarni et al (2020)	467	Whole blood	µg/L	BSID-II: PDI	6 months	RR	late pregnancy	-0.27	-4.11	5.46		
Snoj Tratnik et al (2017)	283	Hair	Ln µg/g	Bayley-III: Fine Motor scale	16 - 20 months	RR		-0.29	-0.59	0.01	0.06	

Snoj Tratnik et al (2017)	283	Hair	Ln µg/g	Bayley-III: Gross Motor scale	16 - 20 months	RR		-0.12	-0.42	0.18	0.44	
Snoj Tratnik et al (2017)	283	Hair	Ln µg/g	Bayley-III: Motor developme nt	16 - 20 months	RR		-1.04	-2.43	0.34	0.14	
Snoj Tratnik et al (2017)	280	Umbilical cord	Ln µg/g	Bayley-III: Fine Motor scale	16 - 20 months	RR		-0.33	-0.66	-0.01	0.04	
Snoj Tratnik et al (2017)	280	Umbilical cord	Ln µg/g	Bayley-III: Gross Motor scale	16 - 20 months	RR		-0.1	-0.41	0.22	0.55	
Snoj Tratnik et al (2017)	280	Umbilical cord	Ln µg/g	Bayley-III: Motor developme nt	16 - 20 months	RR		-1.16	-2.76	0.44	0.15	
Strain et al (2015)	1243	Hair	µg/g	BSID-II: PDI	20 months	RR		0.03			0.68	
Tatsuta et al (2017)	566	Umbilical cord	Ln ng/g	BSID-II: PDI	18 months	RR		-0.12			0.009	Yes
Valent et al (2013)	505	Hair	Ln ng/g	Bayley-III: Motor developme nt	18 months	RR		-0.19			0.616	Yes
Valent et al (2013)	378	Umbilical cord	Ln ng/g	Bayley-III: Motor developme nt	18 months	RR		0.16			0.684	Yes
Wang et al (2019)	265	Umbilical cord	ng/g	Bayley-III: Motor developme nt	20 months	RR		0.124	-0.38	0.63		
Xu et al (2016)	270	Umbilical cord	µg/L	NNNS: Asymmetry	5 weeks	RR	Male	0.10			0.36	Yes
Xu et al (2016)	270	Umbilical cord	µg/L	NNNS: Asymmetry	5 weeks	RR	Female	0.073			0.4	Yes
Xu et al (2016)	270	Umbilical cord	µg/L	NNNS: Handling	5 weeks	RR		-0.02			0.35	Yes
Xu et al (2016)	344	Whole blood	µg/L	NNNS: Asymmetry	5 weeks	RR	Male	-0.13			0.3	Yes

Xu et al (2016)	344	Whole blood	µg/L	NNNS: Asymmetry	5 weeks	RR	Female	0.08			0.43	Yes
Xu et al (2016)	344	Whole blood	µg/L	NNNS: Handling	5 weeks	RR		-0.001			0.98	Yes
Language												
**Study**	**n**	**Exposure**	**Units**	**Outcome**	**Time of outcome**	**Estimate type**	**Notes**	**Estimate**	**LCI**	**UCI**	**P-value**	**High quality**
Barbone et al (2019)	1272	Hair	ng/g	Bayley-III: Expressive Communica tion scale	18 months	RR		0.04	-0.06	0.13		Yes
Barbone et al (2019)	1086	Hair	ng/g	Bayley-III: Language composite	18 months	RR		0.55	0.05	1.05		Yes
Barbone et al (2019)	1075	Hair	ng/g	Bayley-III: Receptive Communica tion scale	18 months	RR		0.12	0.02	0.22		Yes
Barbone et al (2019)	1070	Umbilical cord	ng/g	Bayley-III: Expressive Communica tion scale	18 months	RR		0.01	-0.09	0.11		Yes
Barbone et al (2019)	896	Umbilical cord	ng/g	Bayley-III: Language composite	18 months	RR		0.25	-0.29	0.78		Yes
Barbone et al (2019)	887	Umbilical cord	ng/g	Bayley-III: Receptive Communica tion scale	18 months	RR		0.12	-0.08	0.32		Yes
Barbone et al (2019)	727	Whole blood	ng/g	Bayley-III: Expressive Communica tion scale	18 months	RR		0.13	-0.22	0.48		Yes
Barbone et al (2019)	628	Whole blood	ng/g	Bayley-III: Receptive Communica tion scale	18 months	RR		-0.02	-0.12	0.08		Yes
Daniels et al (2004)	1054	Umbilical cord	µg/g	MCDI: Vocabulary Comprehen sion	15 months	RR		6.1			0.8	Yes

Daniels et al (2004)	1054	Umbilical cord	µg/g	DDST: Language	18 months	RR		0.1			0.9	Yes
Hu et al (2016)	410	Umbilical cord	Ln µg/L	GDS: Language domain	12 months	RR		2.17	-1.88	6.21		Yes
Hu et al (2016)	410	Whole blood	Ln µg/L	GDS: Language domain	12 months	RR		1.92	-3.61	7.46		Yes
Lin et al (2013)	230	Umbilical cord	Hg ≥ 19.78 µg/L	CDIT: Language	24 months	RR		-0.16			NS	
Nisevic et al (2019)	257	Umbilical cord	µg/L	Bayley-III: Language composite	18 months	RR		-0.05			0.74	Yes
Nyanza et al (2021)	429	Whole blood	µg/L	MDAT: Language	6-12 months	aPR		1.05	1.03	1.07		
Oken et al (2008)	341	Erythrocyte	ng/g	PPVT	36 months	RR		-0.4	-0.8	-0.1		Yes
Polanska et al (2013)	303	Hair	µg/g	Bayley-III: Language composite	12-24 months	RR		2.7	-3.9	9.4		
Snoj Tratnik et al (2017)	283	Hair	Ln µg/g	Bayley-III: Language composite	16 - 20 months	RR		-0.01	-2.32	2.3	0.67	
Snoj Tratnik et al (2017)	280	Umbilical cord	Ln µg/g	Bayley-III: Language composite	16 - 20 months	RR		-1.03	-3.46	1.4	0.41	
Valent et al (2013)	505	Hair	Ln ng/g	Bayley-III: Language composite	18 months	RR		0.85			0.11	Yes
Valent et al (2013)	378	Umbilical cord	Ln ng/g	Bayley-III: Language composite	18 months	RR		0.41			0.46	Yes
Wang et al (2019)	236	Umbilical cord	ng/g	Bayley-III: Language composite	19 months	RR		-0.19	-1.02	0.63		

Social development

Study	n	Exposure	Units	Outcome	Time of outcome	Estimate type	Notes	Estimate	LCI	UCI	P-value	High quality
Daniels et al (2004)	1054	Umbilical cord	µg/g	MCDI: Social activity	15 months	RR		-0.2			0.9	Yes

Daniels et al (2004)	1054	Umbilical cord	µg/g	DDST: Social Activity	18 months	RR		0.5			0.8	Yes
Hu et al (2016)	410	Umbilical cord	Ln µg/L	GDS: Social domain	12 months	RR		4.06	0.51	7.62		Yes
Hu et al (2016)	410	Whole blood	Ln µg/L	GDS: Social domain	12 months	RR		0.74	-5.77	4.31		Yes
Lin et al (2013)	230	Umbilical cord	Hg ≥ 19.78 µg/L	CDIT: Social	24 months	RR		-4.39			NS	
Nyanza et al (2021)	429	Whole blood	µg/L	MDAT: Social status	6-12 months	aPR		0.9	0.8	1.1		
Valent et al (2013)	505	Hair	Ln ng/g	Bayley-III: Social-emotional	18 months	RR		1.77			0.11	Yes
Valent et al (2013)	378	Umbilical cord	Ln ng/g	Bayley-III: Social-emotional	18 months	RR		-0.07			0.95	Yes
**General or composite measures of neurodevelopmental functioning**												
**Study**	**n**	**Exposure**	**Units**	**Outcome**	**Time of outcome**	**Estimate type**	**Notes**	**Estimate**	**LCI**	**UCI**	**P-value**	**High quality**
Daniels et al (2004)	1054	Umbilical cord	µg/g	DDST: Total	18 months	RR		0.4			0.9	Yes
Golding et al (2016)	2643	Whole blood	µg/L	DDST-II	18 months	RR		0.49	0.1	0.88	0.01	Yes
Golding et al (2016)	2452	Whole blood	µg/L	DDST-II	30 months	RR		0.23	-0.08	0.53	0.15	Yes
Golding et al (2016)	2394	Whole blood	µg/L	DDST-II	32 months	RR		0.43	0.08	0.78	0.02	Yes
Golding et al (2016)	2721	Whole blood	µg/L	DDST-II	6 months	RR		0.51	0.05	1.0	0.03	Yes
Hu et al (2016)	410	Umbilical cord	Ln µg/L	GDS: Adaptive domain	12 months	RR		4.22	0.77	7.67		Yes
Hu et al (2016)	410	Whole blood	Ln µg/L	GDS: Adaptive domain	12 months	RR		0.65	-4.3	5.59		Yes
Lin et al (2013)	230	Umbilical cord	Hg ≥ 19.78 µg/L	CDIT: Self-help	24 months	RR		3.99			NS	

Lin et al (2013)	230	Umbilical cord	Hg ≥ 19.78 µg/L	CDIT: Whole	24 months	RR		-1.75			NS	
Marques et al (2009)	82	Hair	µg/g	Gesell total developme nt quotient	6 months	RR		-0.27			0.39	
Nyanza et al (2021)	429	Whole blood	µg/L	MDAT: Global neurodevel opment	6-12 months	aPR		1.03	1.01	1.04		
Oken et al (2008)	341	Erythrocyte	ng/g	WRAVMA total	36 months	RR		-0.06	-0.4	0.2		Yes
Suzuki et al (2010)	498	Hair	µg/g	NBAS	3 days	RR		-0.12			<0.05	Yes
Valent et al (2013)	362	Hair	Ln ng/g	Bayley-III: Adaptive behaviour	18 months	RR		0.55			0.57	Yes
Valent et al (2013)	271	Umbilical cord	Ln ng/g	Bayley-III: Adaptive behaviour	18 months	RR		-0.59			0.57	Yes
Wang et al (2019)	172	Umbilical cord	ng/g	NBNA	3 days	RR		-0.07	-0.14	-0.002		
Wu et al (2014)	418	Umbilical cord	µg/L	NBNA	3 days	RR		0.03			0.04	
